# Altered white matter microstructure is associated with social cognition and psychotic symptoms in 22q11.2 microdeletion syndrome

**DOI:** 10.3389/fnbeh.2014.00393

**Published:** 2014-11-11

**Authors:** Maria Jalbrzikowski, Julio E. Villalon-Reina, Katherine H. Karlsgodt, Damla Senturk, Carolyn Chow, Paul M. Thompson, Carrie E. Bearden

**Affiliations:** ^1^Department of Psychiatry and Biobehavioral Sciences, Semel Institute for Neuroscience and Human Behavior, University of California at Los AngelesLos Angeles, CA, USA; ^2^Imaging Genetics Center, Institute for Neuroimaging and Informatics, Keck School of Medicine, University of Southern CaliforniaMarina del Rey, CA, USA; ^3^Center for Psychiatric Neuroscience, The Feinstein Institute for Medical ResearchManhasset, NY, USA; ^4^Division of Psychiatric Research, Zucker Hillside HospitalGlen Oaks, NY, USA; ^5^Psychiatry, Hofstra Northshore-LIJ School of MedicineHempstead, NY, USA; ^6^Department of Biostatistics, School of Public Health, University of California at Los AngelesLos Angeles, CA, USA; ^7^Department of Psychology, University of California at Los AngelesLos Angeles, CA, USA

**Keywords:** DTI, theory of mind, psychosis, schizophrenia, velocardiofacial syndrome, axial diffusivity, radial diffusivity, prodromal

## Abstract

22q11.2 Microdeletion Syndrome (22q11DS) is a highly penetrant genetic mutation associated with a significantly increased risk for psychosis. Aberrant neurodevelopment may lead to inappropriate neural circuit formation and cerebral dysconnectivity in 22q11DS, which may contribute to symptom development. Here we examined: (1) differences between 22q11DS participants and typically developing controls in diffusion tensor imaging (DTI) measures within white matter tracts; (2) whether there is an altered age-related trajectory of white matter pathways in 22q11DS; and (3) relationships between DTI measures, social cognition task performance, and positive symptoms of psychosis in 22q11DS and typically developing controls. Sixty-four direction diffusion weighted imaging data were acquired on 65 participants (36 22q11DS, 29 controls). We examined differences between 22q11DS vs. controls in measures of fractional anisotropy (FA), axial diffusivity (AD), and radial diffusivity (RD), using both a voxel-based and region of interest approach. Social cognition domains assessed were: Theory of Mind and emotion recognition. Positive symptoms were assessed using the Structured Interview for Prodromal Syndromes. Compared to typically developing controls, 22q11DS participants showed significantly lower AD and RD in multiple white matter tracts, with effects of greatest magnitude for AD in the superior longitudinal fasciculus. Additionally, 22q11DS participants failed to show typical age-associated changes in FA and RD in the left inferior longitudinal fasciculus. Higher AD in the left inferior fronto-occipital fasciculus (IFO) and left uncinate fasciculus was associated with better social cognition in 22q11DS and controls. In contrast, greater severity of positive symptoms was associated with lower AD in bilateral regions of the IFO in 22q11DS. White matter microstructure in tracts relevant to social cognition is disrupted in 22q11DS, and may contribute to psychosis risk.

## INTRODUCTION

22q11.2 Microdeletion Syndrome (22q11DS; also known as Velocardiofacial syndrome or DiGeorge Syndrome) is a neurogenetic disorder that carries significantly increased risk for developing psychosis (Pulver et al., 1994; Murphy et al., 1999; Gothelf et al., 2007; Green et al., 2009; Schneider et al., 2014). One prevailing model of psychosis pathogenesis is that of a ‘developmental disconnection’ syndrome, whereby genetic and neurodevelopmental influences lead to structural abnormalities in brain regions critical for cerebral communication (Karlsgodt et al., 2008). This dysconnectivity may be particularly salient during adolescence given that the brain is still developing, which may be related to the emergence of psychotic symptoms in vulnerable individuals (Paus et al., 2008). Thus, studying adolescents and young adults with 22q11DS, a highly penetrant disorder with well-defined genetic etiology, can help us understand how structural dysconnectivity affects a complex psychiatric phenotype (i.e., psychosis).

Diffusion weighted imaging (DWI), which measures the diffusion patterns of water molecules in brain tissue, offers a method to examine structural connectivity between brain regions (Mori and Zhang, 2006). Specifically, fractional anisotropy (FA), a measure derived from diffusion tensor imaging (DTI), indicates the directionality and density of the fiber tracts in a voxel, and has been traditionally viewed as a measure of white matter or myelin integrity (Thomason and Thompson, 2011). FA values are calculated based on the ratio of the longest to shortest axes of diffusion, and values fall between zero and one: zero indicates that the diffusion is isotropic, or unrestricted in all directions, indicating an absence of organized fiber tracts to constrain directionality. A value closer to one means that diffusion occurs more strongly along one axis, suggesting increased fiber organization and white matter integrity. FA is a standard measure of “white matter integrity,” but other DTI indices, such as axial diffusivity (AD) and radial diffusivity (RD), may be more informative regarding the specific nature of the white matter dysfunction (Alexander et al., 2007). For example, AD is a measure of diffusivity along the principal axis, and decreases in AD have been linked with greater axonal damage in rodents (Song et al., 2003; Budde et al., 2008, 2009). RD is an average of the measures of the diffusivities in the two minor axes, and is thought to index the amount of space between axons; accordingly, increased RD has been associated with demyelination in animal models (Song et al., 2002, 2003, 2005). These explanations for FA, AD, and RD are generally accepted, but there may be alternative interpretations regarding the underlying white matter microstructure, particularly where fibers cross (Leow et al., 2009; Zhan et al., 2009). Studying these three indices within a 22q11DS sample and examining their relationships with psychotic symptoms may reveal important information about mechanistic brain changes relevant to the development of psychosis.

Since DTI was introduced in 1994, there have been over 200 articles published on DTI and idiopathic schizophrenia. These studies have revealed disrupted white matter integrity (i.e., FA reductions) in multiple white matter tracts across the phases of illness, including individuals at clinical high-risk (CHR) for psychosis (Karlsgodt et al., 2009; Bloemen et al., 2010; Carletti et al., 2012), first-episode (Peters et al., 2008; Price et al., 2008; Luck et al., 2010; Lee et al., 2013), chronically ill (Friedman et al., 2008; Kong et al., 2011), and medication-naïve schizophrenia patients (Guo et al., 2012; Henze et al., 2012; Liu et al., 2013). The most consistently reported abnormalities are in fronto-temporal and fronto-limbic tracts (Ellison-Wright and Bullmore, 2009; Kuswanto et al., 2012; Samartzis et al., 2014), including the superior longitudinal fasciculus (SLF; Szeszko et al., 2008; Clemm von Hohenberg et al., 2014), one of the largest long-range fiber tracts in the brain which connects the parietal to frontal lobes, and the uncinate fasciculus (Burns et al., 2003; Szeszko et al., 2005; Kawashima et al., 2009; Kitis et al., 2012), a tract which connects regions of the limbic system with orbitofrontal cortex. Increased RD may drive global FA reductions in schizophrenia (Seal et al., 2008; Lee et al., 2013; Scheel et al., 2013; Fitzsimmons et al., 2014) findings which are supported by the post-mortem histopathology literature, which indicates disturbances in the function and structure of oligodendrocytes (Davis et al., 2003; Walterfang et al., 2006), brain cells responsible for the myelination of axons.

A recent review of the existing literature on anomalous white matter development associated with schizophrenia proposes a model that combines the influence of both neurodevelopmental and neuroprogressive influences on white matter in schizophrenia (Peters and Karlsgodt, 2014). In this model, both early disruption of white matter development, particularly during adolescence, paired with later white matter microstructural changes due to disease chronicity, medication effects, or later onset progressive changes result in the white matter abnormalities seen in schizophrenia (Peters and Karlsgodt, 2014). Regarding disruption of white matter during adolescence, Karlsgodt et al. (2009), found that youth at clinical high risk for developing psychosis failed to show the typical age-associated increased of FA in the medial temporal lobe in comparison to typically developing controls. There is preliminary evidence that 22q11DS participants also show a disrupted trajectory of white matter development; one research group found that an overall measure of total mean FA increased in controls, but that this relationship was not observed in 22q11DS participants (Ottet et al., 2013a). However, age-associated disruptions in 22q11DS have not been examined in specific white matter tracts.

While many studies have shown that decreased FA is associated with cognitive functioning in patients with idiopathic schizophrenia (Yan et al., 2012; Liu et al., 2013; Nestor et al., 2013; Roalf et al., 2013), to our knowledge only one DTI study of CHR youth has examined the relationship between DTI measures and social processes. In this longitudinal study, lower FA in the medial temporal lobe and inferior longitudinal fasciculus (ILF) predicted a drop in social functioning in CHR participants 15 months later (Karlsgodt et al., 2009). Given that many brain regions affected in schizophrenia are also implicated in social cognition (Pinkham et al., 2003) and a prior study by our laboratory found that a measure of social cognition (Theory of Mind) was the most significant predictor of psychotic symptoms in 22q11DS (Jalbrzikowski et al., 2012), we aimed to examine whether white matter microstructural integrity is linked to social-cognitive dysfunction in 22q11DS.

As in idiopathic schizophrenia and those at clinical high risk, white matter integrity may also be disrupted in multiple brain regions in 22q11DS, but findings are mixed. Three cross-sectional studies of children with 22q11DS found lower FA in the SLF (Barnea-Goraly et al., 2003; Sundram et al., 2010; Villalon-Reina et al., 2013), complementing existing schizophrenia findings (Federspiel et al., 2006; Szeszko et al., 2008; Karlsgodt et al., 2009; Clemm von Hohenberg et al., 2014). The largest existing DTI study of 22q11DS to date (33 22q11DS and 16 unaffected siblings, mean age: 18.0 years), found bilateral FA reductions in the uncinate fasciculus (Radoeva et al., 2012), which is also consistent with the idiopathic schizophrenia literature (Burns et al., 2003; Szeszko et al., 2005; Kawashima et al., 2009; Kitis et al., 2012). Lower FA in the ILF and splenium of the corpus callosum has also been observed in both 22q11DS (Villalon-Reina et al., 2013) and idiopathic schizophrenia (Gasparotti et al., 2009; Liu et al., 2013). In adults with 22q11DS, disruption in other white matter tracts has been observed in multiple brain regions, including the parietal (da Silva Alves et al., 2011; Kikinis et al., 2012) and parahippocampal regions (da Silva Alves et al., 2011). Preliminary evidence suggests that white matter microstructural abnormalities in 22q11DS may be driven by reduced axonal coherence (Kikinis et al., 2012, 2013; Radoeva et al., 2012).

However, other studies have found that, compared to healthy controls, 22q11DS participants have *increased* FA in the splenium and genu of the corpus callosum (Barnea-Goraly et al., 2003; Sundram et al., 2010), the inferior fronto-occipital fasciculus (IFO) and SLF (Simon et al., 2005) and portions of the corona radiata (Sundram et al., 2010). These studies did not investigate the component measures that comprise FA (AD and RD), so it is not clear what was contributing to these increases. Additionally, given that most current publications have <20 patients in their sample, larger sample sizes are warranted, in order to clarify the nature of white matter pathology in 22q11DS.

Notably, there is some evidence for a relationship between white matter integrity and social behavior in 22q11DS. Specifically, in a combined analysis of 22q11DS participants and controls, increased AD in the posterior corona radiata, SLF, and IFO was related to better social skills (Radoeva et al., 2012). Additionally, reduced FA in frontal, cingulate, and temporal regions was associated with increased psychotic symptom severity in adults with 22q11DS (da Silva Alves et al., 2011). However, no study has simultaneously examined laboratory-based measures of social cognition (e.g., Theory of Mind, emotion recognition) and psychotic symptoms and their relationship to DTI measures in individuals with 22q11DS.

Our study had three main goals: (1) to examine group differences between 22q11DS participants and controls on multiple DTI measures (e.g., FA, AD, and RD) via a whole-brain and ROI-based approach; (2) to explore where there are age-associated disruptions in these DTI metrics in 22q11DS patients versus typically developing controls, and (3) to relate positive symptoms and social cognition performance to measures of white matter microstructure in 22q11DS and controls. First, based on prior work (Sundram et al., 2010; Radoeva et al., 2012), we expected to find lower FA in long-range fiber tracts in 22q11DS relative to controls, including the SLF and uncinate fasciculus, which would be driven by abnormal AD (Kikinis et al., 2012; Radoeva et al., 2012). Based on previous literature in CHR youth and 22q11DS (Karlsgodt et al., 2009; Ottet et al., 2013a), we hypothesized that 22q11DS youth would fail to show the typical age-associated increases in FA observed in typically developing controls (Simmonds et al., 2014). We also hypothesized that integrity of the uncinate fasciculus, a fronto-limbic white matter tract relevant to social cognition and previously shown to be disrupted in both patients with 22q11DS and idiopathic schizophrenia (Barnea-Goraly et al., 2003; Szeszko et al., 2005), would be associated with positive symptom severity and social cognition in 22q11DS.

## MATERIALS AND METHODS

### PARTICIPANTS

The initial sample consisted of 76 participants (10–26 years old, 40 22q11DS, and 36 controls). DTI data from 11 participants (4 22q11DS, 7 controls) were excluded due to poor image quality or severe motion/scanning artifacts. Thus, the final sample consisted of 65 participants (36 22q11DS, 29 controls, **Table 1**).

**Table 1:** Demographic and clinical characteristics of study participants.

	22q11.2 Microdeletion Syndrome (22q11DS) Participants (*n* = 36)	Healthy Comparison Participants (*n* = 29)	
Age (years, ±SD)	16.3 (4.3)	15.5 (3.8)	*p* = 0.46
Participant Education (years, ±SD)	9.2 (3.3)	9.4 (3.9)	*p* = 0.78
Parental Education (years, ±SD)	16.3 (2.3)	15.5 (3.1)	*p * = 0*.25*
Gender (*N*, % female)	25 (69%)	14 (48%)	*p* = 0.14
Race (Asian/African American/Caucasian/Multiple)	0/1/32/3	1/3/20/5	*p* = 0.21

Psychotic Disorder Diagnosis (*N*, %)	4 (11%)	NA	
SIPs Positive Symptoms (mean, ±SD)	6.6 (7.1)	0.7 (1.3)	*p* < 0.001
SIPs Negative Symptoms	8.1 (6.3)	1.0 (1.8)	*p* < 0.001
SIPs Disorganized Symptoms	4.6 (4.8)	0.5 (0.9)	*p* < 0.001
SIPs General Symptoms	4.9 (5.0)	0.9 (1.4)	*p* < 0.001
Psychotropic Medication (*N*, None/Antipsychotics/Antidepressants)	26/4/6	NA	
WASI IQ Score	78.1 (14.8)	107.4 (17.6)*^a^*	*p* < 0.001

*^a^IQ score available for 27 controls.*

22q11DS participants all had a molecularly confirmed diagnosis of 22q11.2 deletion syndrome and were recruited from an ongoing longitudinal study at the University of California, Los Angeles (UCLA). Typically developing healthy controls were also recruited from this study. Exclusion criteria for all study participants were: neurological or medical condition disorder that might affect performance, insufficient fluency in English, and/or if they endorsed substance or alcohol abuse and/or dependence within the past six months. Controls also must not meet criteria for any major mental disorder, with the exception of attention deficit-hyperactivity disorder (ADHD) or past episode of depression, based on information gathered during the Structured Clinical Interview for DSM-IV Axis I Disorders (First et al., 2002). Three controls in our sample had a past single episode of depression, and none of our controls had a diagnosis of ADHD. We did not exclude 22q11DS patients with comorbid medical conditions, given that these conditions are characteristic of the disorder.

All participants gave verbal and written informed consent. Participants under the age of 18 years provided written assent, while their parent or guardian completed written consent. The UCLA Institutional Review Board (IRB) approved all study procedures and informed consent documents.

### MEASURES

#### Structured interview for prodromal syndromes

A master’s level trained clinician assessed all participants on the positive, negative, disorganized, and general symptom scales from the Structured Interview for Prodromal Syndromes (SIPS, McGlashan et al., 2001). Symptoms on these scales are rated from 0 to 6, with zero representing an absence of symptoms and six referring to an extremely severe level of symptoms. This measure has shown excellent inter-rater reliability (Miller et al., 2003; Meyer et al., 2005). All raters demonstrated good to excellent inter-rater reliability for symptom ratings, with kappa values ranging from 0.85 to 1.00. For the purposes of this study, we used the sum of the positive SIPS symptom scores as a dimensional measure of psychotic symptom severity. This measure encompasses a range of symptom severity, including sub-threshold (prodromal), and fully psychotic symptoms.

#### Social cognition tasks

Study participants received the Penn Emotion Recognition Test (ER40), a computerized emotion identification task in which 40 color photographs of adult faces, varying in race and gender, are randomly presented (Kohler et al., 2000). Participants were asked to identify the emotion of each face (happy, sad, anger, fear, or no emotion) and were given as long as needed to respond (total maximum score = 40, each emotion presented eight times). This measure has shown adequate construct validity and test–retest reliability (Carter et al., 2009), and has been previously used with adolescents (Schenkel et al., 2007; Roddy et al., 2012) and 22q11DS participants (Goldenberg et al., 2012; Gur et al., 2014).

All participants were administered Part 3 of The Awareness of Social Inference Test (TASIT; McDonald et al., 2003). The TASIT is a computerized task believed to assess one’s ability to comprehend the intentions of others, particularly how one comprehends white lies or sarcasm. The task consists of 16 vignettes (each lasting between 15 and 60 s), eight of which show an individual telling a lie, while the other eight display an interaction in which someone uses sarcasm. After viewing each vignette, an assessor asked the participant four questions related to the scene: (1) what someone is doing to another person in the scene, (2) what someone is trying to say to the other person, (3) what one of the individuals in the scene is thinking, and (4) what one of the characters in the vignette is feeling. After task completion, an overall score was calculated (maximum = 64). The TASIT has shown adequate reliability and validity with brain-injured patients (McDonald et al., 2006), and has been used with adolescents at CHR for psychosis, along with first-episode and chronic patients with schizophrenia (Green et al., 2012).

### IMAGE ACQUISITION

All scanning was carried out on an identical Siemens 3 Tesla Tim Trio MRI scanner at either the UCLA Brain Mapping Center (BMC; 22q11DS = 15, controls = 16) or at the Center for Cognitive Neuroscience (CCN; 22q11DS = 21, controls = 13). The age distribution for both 22q11DS patient and control subjects did not differ across scanner locations. There were, however, significantly more female 22q11DS participants compared to controls scanned at the CCN (*p* = 0.009); however, the gender distribution was similar for all other comparisons (Supplementary Table S1). DTI and structural MRI protocols were identical at both scanner locations. Specifically, measures of brain structure were obtained using T1-weighted anatomical images acquired with an MPRAGE sequence with the following acquisition parameters: TR/TE/TI = 2300/2.91/900; flip angle = 9°; slice thickness = 1.20 mm, with a 240 × 256 acquisition matrix. A diffusion-weighted, spin-echo echo-planar imaging scan was collected using these parameters: 64 diffusion gradient directions, TR/TE = 7100/93 ms; FOV = 190 mm × 190 mm; 96 × 96 matrix; slice thickness = 2.0 mm; b-value = 1000 s/mm^2^ and one non-diffusion sensitized volume was also acquired (*b*-value = 0/s/mm^2^), which we will call the *b*0 image.

### IMAGE ANALYSIS

The T1-weighted images were skull-stripped using Brainsuite’s surface extraction tool (BSE), and then manually edited to remove any remaining non-brain tissue. The skull-stripped T1 images were then linearly aligned using FSL’s “FLIRT” (with 6 degrees of freedom) to a common space (MNI-ICBM 152 non-linear sixth generation) with 1mm isotropic voxels and a 182 × 182 × 182 voxel matrix. For the DWI scans, we removed non-brain regions from the T2-weighted weighted *b0* image with FSL’s brain extraction tool (BET) and the resulting mask was then applied to the remaining 64 volumes. We then corrected the images for eddy current distortion by using FSL’s eddy correct tool. The original gradient vectors were rotated using the linear rotation and translation matrix. Then, each individual’s eddy corrected DWI volumes were linearly aligned (9 degrees of freedom) to the corresponding skullstripped T1 image in the MNI-ICBM 152 standard space. The gradient vectors were once more adjusted by using the resulting linear rotation and translation matrix of this transformation. To compensate for EPI-induced susceptibility artifacts, the *b0* image was non-linearly registered in three directions to the T1 structural scan in the MNI-ICBM 152 space (Leow et al., 2005), by using an elastic regularizer (with a weight of 0.0001) and a cost function based on mutual information. We then applied the derived non-linear vector fields to each of the 64 diffusion-weighted volumes. We then fit diffusion tensors at each voxel in the EPI-corrected DWI volumes by subject using DTIFit (FMRIB’s Diffusion Toolbox). Tensor derived indices such as FA, AD, and RD, were then calculated for the whole brain. We applied the least weighted squares regression for the tensor calculation, which does not rely upon the assumption of homoscedasticity, and has been suggested as a preferred method over an ordinary least squares approach, which is typically used (Jones et al., 2013).

Group maps were then created using FSL’s Tract-Based Spatial Statistics (TBSS). TBSS is a rigorous registration approach, which is imperative for comparisons in which tract shape or volume is likely to differ between groups. Because our sample included children, we aligned each FA map to each other and identified the most representative scan from our sample, which was then used as the target image. All images were then aligned to the target FA image through non-linear registration. All images are merged into a single 4D image and non-linear registration is used to create an FA “skeleton” based on the center of all of the tracts common to the entire group. Data are then projected from the center of each subject’s tracts onto the skeleton for group comparison. This method ensures that statistics are only applied in regions where data exist for all subjects, and maximizes the likelihood that the pooled data originate from the center of a tract in every subject.

To characterize group differences between 22q11DS and controls at the voxel-wise level, we first used a whole-brain based approach. In this approach, we were not restricted to specific areas of interest and could identify nuanced abnormalities that may be obscured by the examining only regions of interest (ROI). Given the variability in 22q11DS findings, we decided to first take an unbiased approach. We also implemented a ROI analysis to confirm our whole-brain analysis, identify age-associated changes in known white matter tracts between 22q11DS and controls, and determine whether DTI indices in specific tracts are related to psychotic symptoms and measures of social cognition in 22q11DS participants and controls.

**FIGURE 1:**
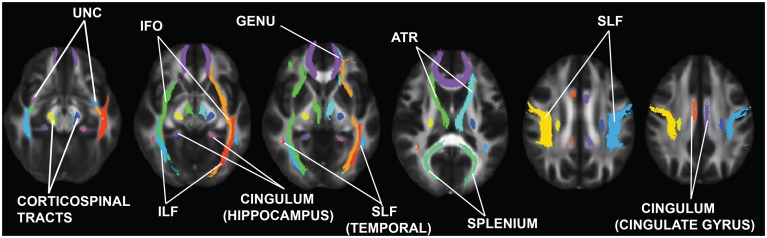
**Regions of interest (ROIs) examined in this study.** These ROIs were obtained from the Johns Hopkins University Probabilistic Tractography atlas (Wakana et al., 2004; Mori and Zhang, 2006). UNC, uncinate fasciculus; ILF, inferior longitudinal fasciculus; IFO, inferior fronto-occipital fasciculus; SLF, superior longitudinal fasciculus; ATR, anterior thalamic radiations.

For the ROI-based approach, 20 regions per hemisphere were identified and determined based on the John Hopkins University probabilistic tractography atlas (**Figure 1**; Wakana et al., 2004; Mori et al., 2005) and then customized based on the TBSS skeleton for the current study. Each subject’s FA, AD, and RD skeleton was masked using each of the ROIs. Then average FA, AD, and RD were calculated and extracted for that segment of the skeleton for each individual.

### STATISTICAL ANALYSES

Statistical analyses were performed using SPSS software v. 21 (Chicago, IL, USA). Correction for multiple comparisons was conducted using publicly available R software (R Core Team, 2014; command p.adjust{stats}). We compared demographic characteristics between groups using independent samples *t*-tests for continuous variables and chi square test for categorical variables. To test for cross-scanner differences, for all DTI measurements we first conducted a univariate analysis of covariance (ANCOVA) for each identified region in each hemisphere, with scanner location as the between-group factor and group as a covariate.

#### Whole-brain analyses

To compare FA, AD, and RD across the entire skeleton in 22q11DS participants vs. controls, we conducted a non-parametric permutation analysis using the ‘randomize’ tool in FSL. We performed 10,000 permutations using the Threshold Free Cluster Environment (TFCE), which is a rigorous method that identifies “clusters” in the data without having to predefine the clusters (Smith and Nichols, 2009). Demeaned age and sex and scanner location were included in the model as covariates and group was identified as the between-groups subjects factor. We corrected for multiple comparisons using family-wise error rate (FWE).

#### Region of interest analyses

All neuroanatomic measures were first examined for normality using the Kolmogorov–Smirnov and Shapiro–Wilk tests; none were found to violate the assumptions of normality. To compare putative indices of white matter integrity (FA), and its sub-components, AD and RD, respectively, in 22q11DS vs. controls, we conducted an ANCOVA for each identified region in each hemisphere, with diagnosis (22q11DS vs. control) as the between subjects factor and sex, age, and scanner location as covariates. For these analyses, due to the large number of comparisons (60), false discovery rate (FDR) *q*-values were estimated using R software.

#### Age × diagnosis interactions

To address whether the relationship between age and DTI measures differed between groups, we first visually examined the scatterplots of age vs. all DTI measures. Being mindful of multiple comparisons, we only wished to conduct statistical tests where the data suggested a possible interaction effect. For any DTI measure for which visual inspection suggested an age^∗^group interaction we then added the age^∗^group interaction term to the original ANCOVA models (in addition to group, age, sex, and scanner location). This resulted in a total of six additional analyses: FA in bilateral regions of the ILF, and SLF, and RD in bilateral regions of the ILF. For any significant age^∗^group interaction, we followed up by examining Pearson correlations between age and the DTI measure within groups. Following a visual inspection of the age^∗^group interactions, we confirmed that the other regions did not show any significant interactions (all *p* > 0.14). Additionally, we ran a Pearson correlation to examine the relationship between age and psychotic symptoms in the 22q11DS sample.

#### Association of DTI measures with social cognition and clinical symptoms

To explore the relationships of DTI measures with positive symptoms and social cognition tasks, we conducted correlational analyses for regions that showed significant group differences between 22q11DS participants and controls. Residuals were calculated from each DTI variable examined, after regressing out the effects of age, sex, and scanner. We also calculated residuals for the clinical and social cognition variables, regressing out the effects of age, sex, and scanner location. Next, we conducted Pearson correlations (corresponding to partial correlations) between each neuroanatomic brain region and residualized total positive symptoms. Due to the restricted range of positive symptoms in controls, we conducted this analysis only in 22q11DS patients. These analyses were then repeated within each group (22q11DS participants and controls), to investigate the relationship between DTI indices and social cognition performance. FDR *q*-values were estimated using R software. In regions that showed significant relationships between DTI indices and social cognition variables, we conducted a secondary analysis in which we also regressed out the effects of global cognitive abilities (WASI IQ score) and re-ran the Pearson correlations.

In regions that showed a significant relationship with overall ER40 performance, we conducted secondary analyses, focusing on Pearson correlations between the DTI measure and performance on each individual emotion (happy, sad, fear, anger, no emotion), particularly since a previous publication from our laboratory showed differential impairment in emotion recognition performance in 22q11DS (Jalbrzikowski et al., 2012).

#### Association between axial and radial diffusivities

It is known that RD and AD make up the measure of FA; however, it is not clear if these two measures are related to each other in white matter tracts of interest. To evaluate the relationship between measures of AD and RD in each group, we correlated the residuals (regressing out effects of age, scanner location, and sex) for each DTI AD variable with the residuals for the corresponding DT RD variable in 22q11DS patients and controls separately. To directly compare the strength of correlations between the two groups, a Fisher r-to-z transformation was conducted. FDR *q*-values were estimated using R software.

## RESULTS

As shown in **Table 1**, 22q11DS patient and control groups did not significantly differ in any of the demographic factors (all *p-*values ≥0.14).

### EFFECTS OF SCANNER LOCATION

We found that scanner location had a significant effect on multiple DTI measures, with significant *q*-values ranging from 0.04 to 0.000001 (see Supplementary Table S2 for more detailed information). These effects were consistent across regions: FA was consistently higher and AD and RD consistently lower in ROI’s showing group differences. As such, all ANCOVAs included scanner site as a covariate.

### GROUP DIFFERENCES: WHOLE BRAIN RESULTS

**FIGURE 2:**
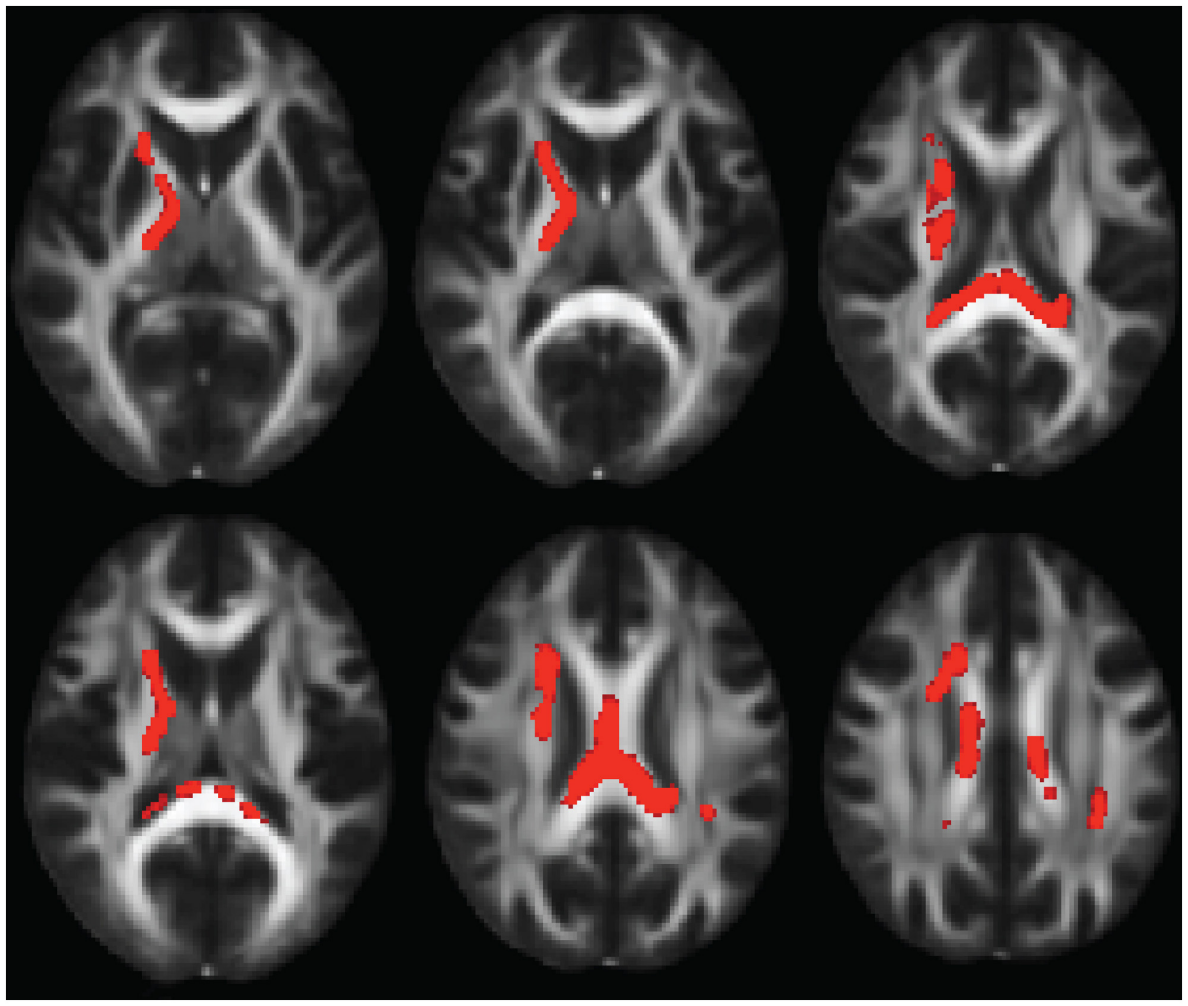
**Multiple slices of brain regions in FSL showing results of whole-brain FA analysis, indicating FA increases in 22q11.2 Microdeletion Syndrome (22q11DS) vs. controls.** Results are shown in the axial view and overlaid on the standard FMRI58 FA template.

**FIGURE 3:**
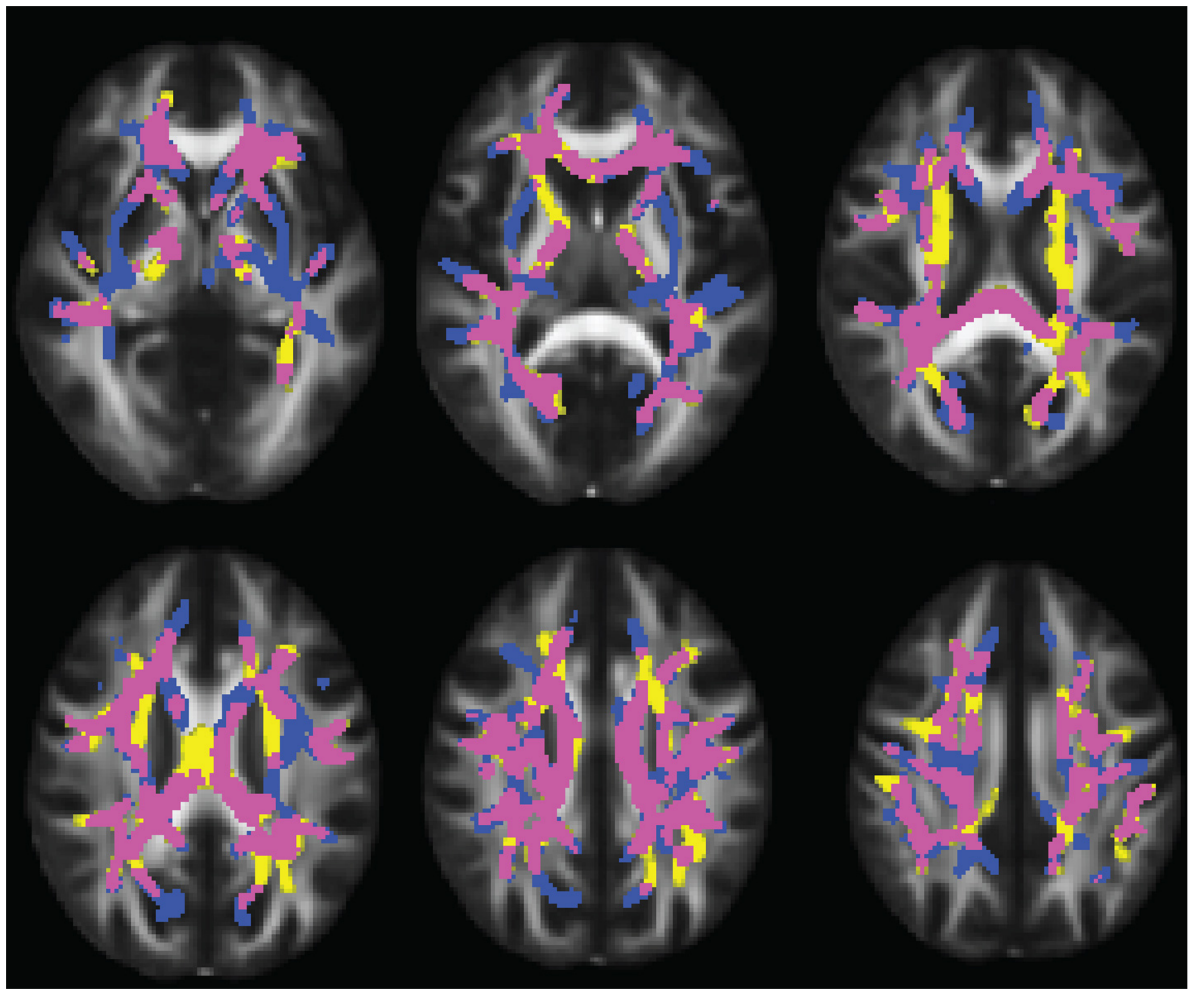
**Figure showing overlap between regions that have reduced axial diffusivity (AD) and radial diffusivity (RD) in 22q11DS.** Regions that share abnormalities in AD and RD are in yellow, regions that have reduced AD only are shown in pink, and regions that have reduced RD only are in blue. Results are shown overlaid on the standard FMRI58 FA template.

Compared to typically developing controls, 22q11DS showed significantly higher FA in multiple regions (**Figure 2**). Most of these significant differences were in the right hemisphere, and included the posterior limb of the internal capsule and the superior and posterior corona radiata. FA was higher in 22q11DS subjects in the body of the corpus callosum and in a small region of the left SLF. In these analyses, compared to typically developing controls, 22q11DS did not show decreased FA in any regions. There were also widespread reductions in AD and RD in 22q11DS in comparison to typically developing controls (**Figure 3**). The body and splenium of the corpus callosum, bilateral regions of ATR, and bilateral portions of the SLF and ILF showed reductions in both AD and RD in 22q11DS. Reductions that were specific to AD (but not observed for measures of RD) in 22q11DS were found in the bilateral IFO, while reductions that were specific to RD in 22q11DS were in the left superior corona radiata and upper regions of the corticospinal tract. When we plotted the overall raw mean FA values for regions that showed significant differences between 22q11DS and controls in the whole-brain analysis (i.e., right posterior limb of the internal capsule, right superior and posterior corona radiata, body of corpus callosum, and a small portion of the left SLF), we saw that FA was consistently higher in 22q11DS participants than controls, regardless of age (Supplementary Figure S1). Thus, the increased FA observed at the whole brain level in 22q11DS does not appear to be the result of an age × group interaction.

### GROUP DIFFERENCES: REGION OF INTEREST ANALYSES

**FIGURE 4:**
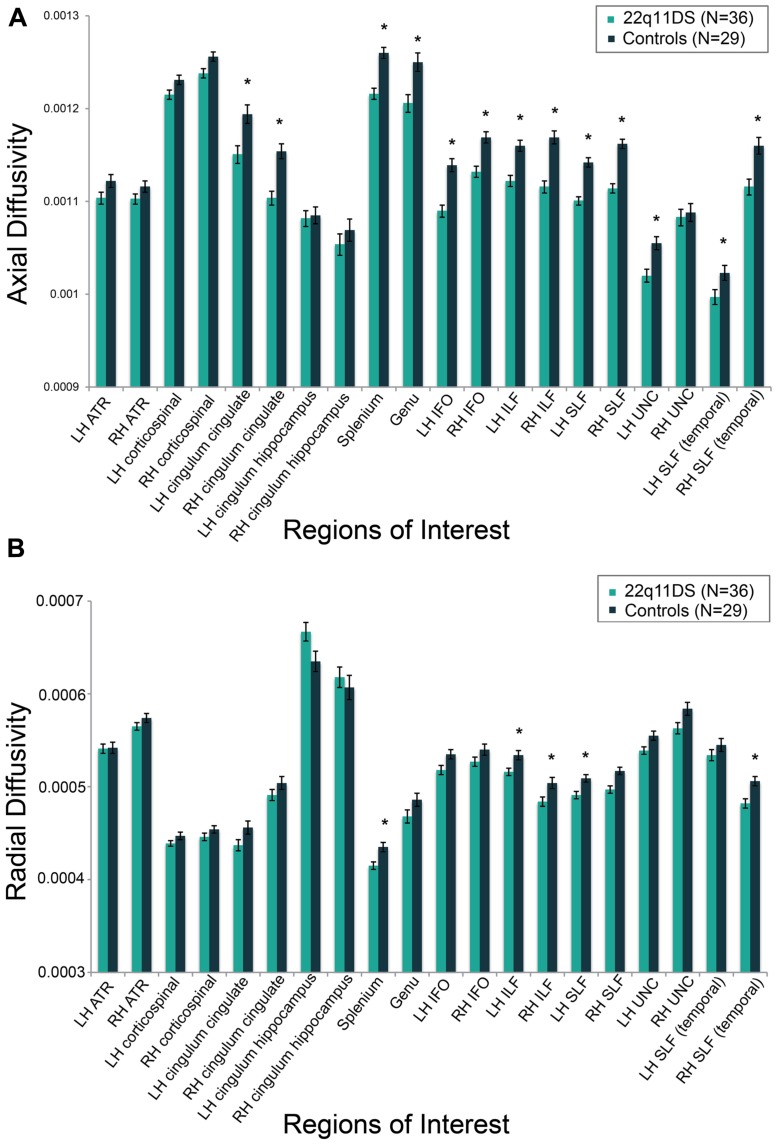
**Group differences between individuals with 22q11DS versus typically developing controls on measures of **(A)** axial diffusivity (AD) and **(B)** radial diffusivity (RD).** AD and RD values are marginal means adjusted for covariates of scanner site, age, and gender. *q < 0.05.

Results for analyses of group differences between 22q11DS vs. controls in measures of FA, AD, and RD within specific white matter tracts are presented in Supplementary Table S3. FA in the region of the left cingulum bundle proximal to the hippocampus was lower in 22q11DS in comparison to typically developing controls. AD was lower in bilateral regions of the anterior portion of the cingulum bundle, IFO, ILF, and SLF. Also, compared to controls, 22q11DS participants had lower AD in the left uncinate fasciculus and the splenium and genu of the corpus callosum. The greatest effect sizes were observed for AD in the SLF (partial η*^2^* for right and left = 0.40; see Supplementary Table S3). RD in the splenium of the corpus callosum and bilateral regions of the ILF and SLF was significantly reduced in 22q11DS in comparison to typically developing controls. AD and RD results are shown in Figures 4A,B, respectively.

All regions that showed significant FA or AD differences remained statistically significant (*p* < 0.05) or approached significance (*p*≤ 0.10) when the total sample was broken into subgroups according to scanner location (BMC, CCN). For measures of RD, differences in the primary tract of the right SLF remained statistically significant (*p* ≤ 0.05) and approached significance in the temporal portion of the SLF (*p* ≤ 0.07) when the sample was divided into sub-groups according to scanner. However, for all other measures of RD that were statistically significant when the whole group was examined (i.e., splenium of corpus callosum, left and right ILF, left SLF) these results only remained statistically significant when comparing 22q11DS vs. controls in the BMC scanner subgroup. Results are presented in Supplementary Table S4.

### AGE × DIAGNOSIS INTERACTIONS

**FIGURE 5:**
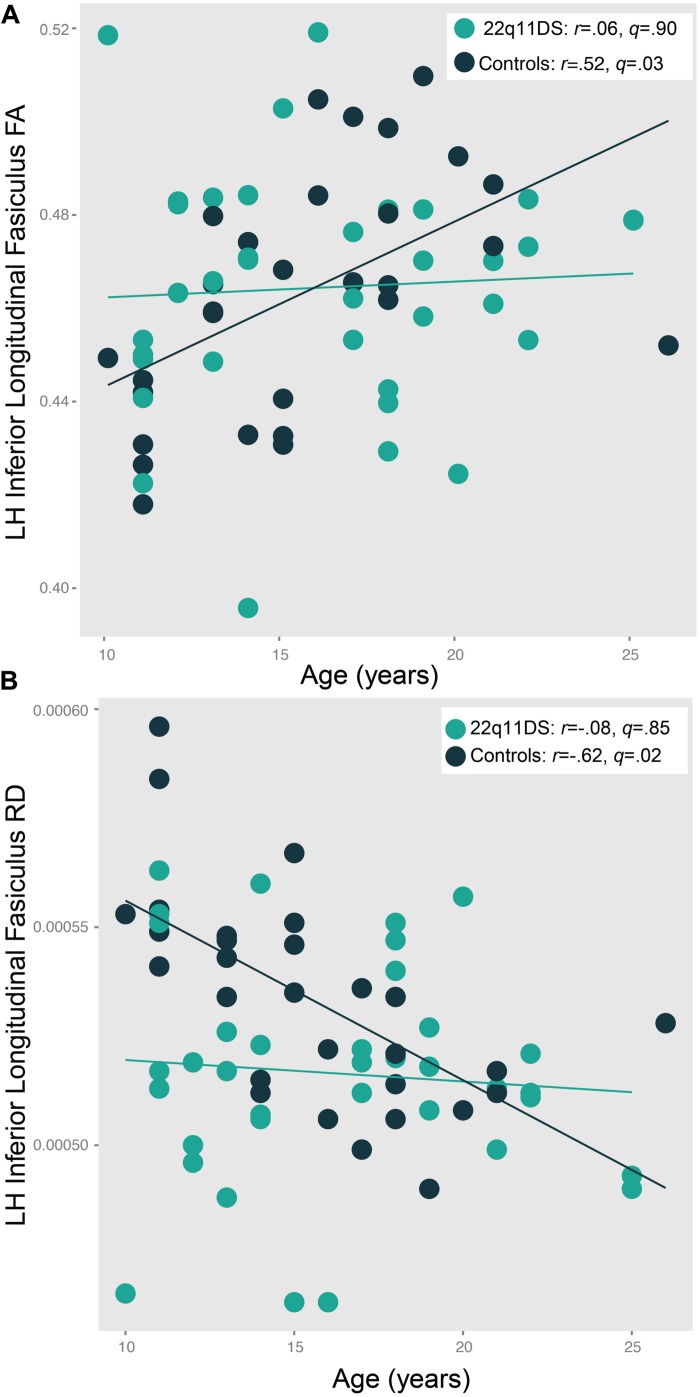
**Patterns of age-associated changes in **(A)** fractional anisotropy (FA) and **(B)** radial diffusivity (RD) in 22q11DS participants versus typically developing controls in the left inferior longitudinal fasciculus (ILF).** Controls show the expected pattern of increased FA and decreased RD with increasing age, which is not observed in 22q11DS patients [*F*(5,59) = 4.2, *p* = 0.04 and *F*(5,59) = 5.8, *p* = 0.02, respectively].

There were significant age^∗^group interactions for left ILF FA [*F*(5,59) = 4.2, *p* = 0.04] and RD [*F*(5,59) = 5.8, *p* = 0.02; **Figure 5**]. In controls, ILF FA values increased with increasing age (*r* = 0.52, *q* = 0.03). However, this relationship was not present in 22q11DS participants (*r* = 0.06, *q* = 0.90). In the left ILF, controls showed decreasing RD with increasing age (*r* = -0.62, *q* = 0.02), but again, this pattern was not seen in 22q11DS (*r* = -0.08, *q* = 0.85). Of note, we did not find a significant relationship with age and psychotic symptoms in 22q11DS (*r* = 0.19, *p* = 0.30).

### RELATIONSHIPS BETWEEN DTI INDICES AND POSITIVE SYMPTOMS IN 22q11DS

**FIGURE 6:**
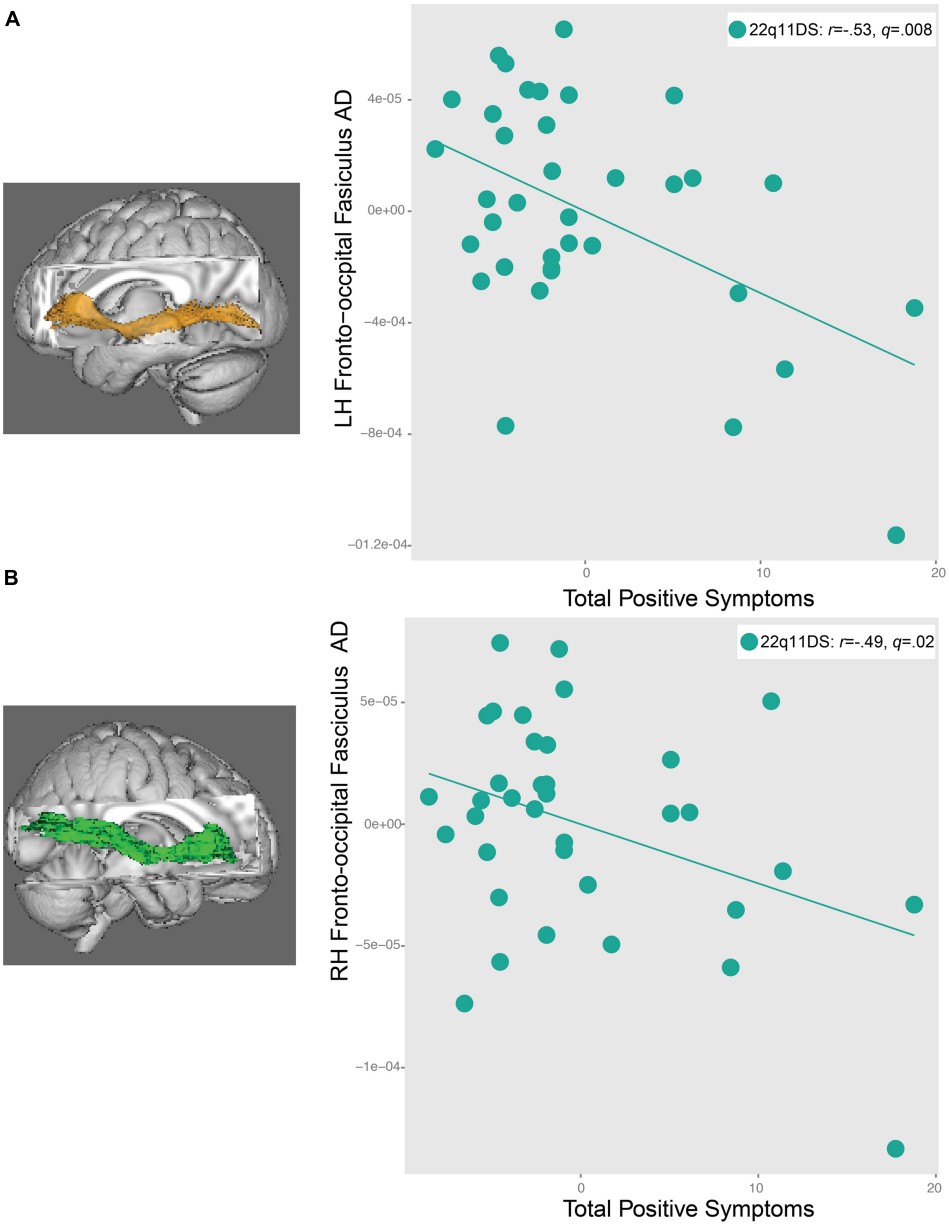
**Relationship between total positive symptoms and axial diffusivity (AD) in the **(A)** left inferior longitudinal fasiculus (IFO) and **(B)** right IFO in 22q11DS participants.** As AD in the left and right IFO decreases, positive symptom severity increases.

Significant relationships were observed between AD in the IFO bilaterally and positive symptoms in 22q11DS (left: *r* = -0.53, *q* = 0.008, right: *r* = -0.49, *q* = 0.02, **Figure 6**). In both regions, decreased AD was associated with greater severity of positive symptoms. There were no relationships between FA and RD and positive symptoms in 22q11DS. When effects of IQ were regressed out, and the significant correlations were re-run, all correlations remained significant (Supplementary Table S5A).

### RELATIONSHIPS OF DTI INDICES WITH SOCIAL COGNITION

**FIGURE 7:**
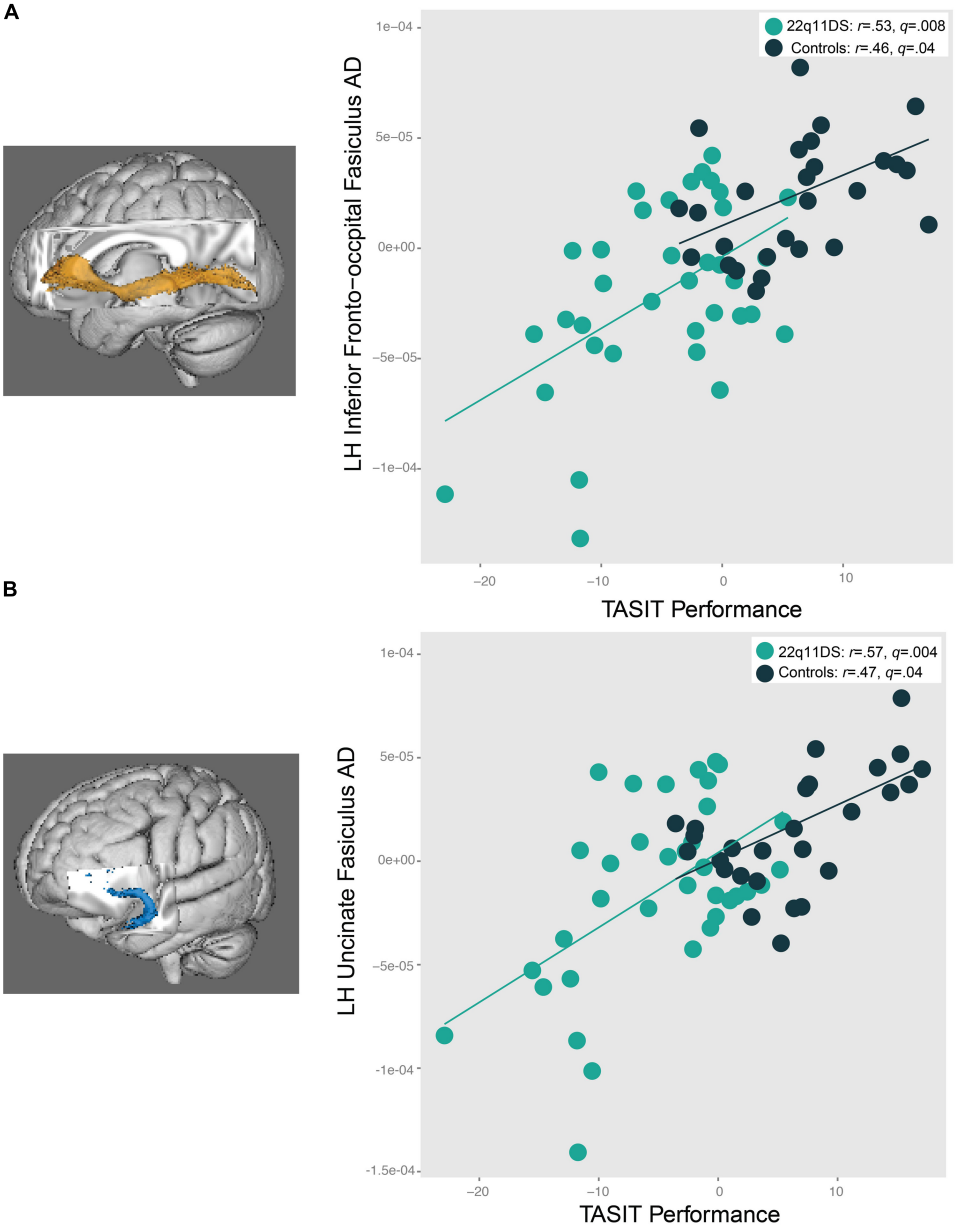
**Relationship between social cognition performance and AD in the **(A)** left IFO and **(B)** left uncinate fasciculus (UNC).** In both 22q11DS participants and controls, as AD in the left IFO and UNC increases, social cognition task performance improves.

As seen in **Figure 7**, increased AD in the left IFO (22q11DS: *r* = 0.53, *q* = 0.008; controls: *r* = 0.46, *q* = 0.04) and left uncinate fasciculus (22q11DS: *r* = 0.57, *q* = 0.004, controls: *r* = 0.47, *q* = 0.04) was associated with better performance on the TASIT in both 22q11DS participants and controls. A similar pattern of results was observed for the relationship between ER40 performance and the left IFO (*r* = 0.47, *q* = 0.02) and left uncinate fasciculus (*r* = 0.45, *q* = 0.03) in 22q11DS, but not in controls (left IFO: *r* = 0.11, *q* = 0.8, left uncinate fasciculus: *r* = -0.07, *q* = 0.8). Controls also showed a significant associations between increased AD in the SLF bilaterally (left SLF: *r* = 0.59, *q* = 0.008; right SLF: *r* = 0.61, *q* = 0.008) and ILF bilaterally (left ILF: *r* = 0.55, *q* = 0.02; right ILF: *r* = 0.47, *q* = 0.04) with better social cognition performance on the TASIT. However, relationships between SLF and ILF AD and TASIT performance were not observed in 22q11DS participants (all *q* > 0.7). When effects of IQ were regressed out, and the significant correlations were re-run, all correlations remained significant for both 22q11DS participants and controls, aside from relationships between social cognition performance and the ILF regions for controls (Supplementary Tables S5B,C).

We conducted secondary analyses of AD measures in the left IFO and left uncinate fasciculus (the only regions that showed a significant relationship with overall ER40 performance) and task performance for specific emotions in 22q11DS. These results indicate that the only emotion that significantly correlated with these two measures was fear, with better recognition of fear associated with increased AD in both regions in 22q11DS (left IFO: *r* = 0.46, *p* = 0.005; left uncinate fasciculus: *r* = 0.38, *p* = 0.02).

### STRENGTH OF LINEAR RELATIONSHIPS BETWEEN MEASURES OF AXIAL AND RADIAL DIFFUSIVITY

Results of these analyses in 22q11DS patients and controls are presented in **Table 2**. Compared to controls, 22q11DS participants showed a significantly stronger linear relationship between AD and RD in the right cingulum bundle (Z = 2.84, *q* = 0.03), the genu (*Z* = 3.2, *q* = 0.01), and the right uncinate fasciculus (*Z* = 2.76, *q* = 0.03). 22q11DS showed significant positive relationships between RD and AD in the right ATR, bilateral regions of the IFO, the right SLF, left uncinate fasciculus, but controls did not show significant relationships in these regions. However, compared to controls, 22q11DS patients did not show a statistically stronger linear relationship in these regions. We found that both controls and patients showed significant positive linear relationships between RD and AD in the left ATR and left cingulum bundle in the hippocampal region. In all cases where there was a linear relationship between AD and RD, increased AD was associated with increased RD.

**Table 2:** Correlations between axial and radial diffusivity (RD) for ROIs within white matter tracts and Fisher *r*-to-*Z* transformation results comparing the correlations of participants with 22q11.2 deletion syndrome versus typically developing controls.

Region	Correlations		Fisher *r*-to-*Z* transformation	
	22q11DS (*N* = 36)	Controls (*N* = 36)	Z	*q*-value
Left anterior thalamic radiations	*r* = 0.75 *q* = 9.1e-6	*r* = 0.57 *q* = 0.01	1.24	0.41
Right anterior thalamic radiations	*r* = 0.70 *q* = 0.00007	*r* = 0.46 *q* = 0.06	1.41	0.35
Left corticospinal tracts	*r* = -0.10	*r* = 0.62	-2.38	0.07
	*q* = 0.81	*q* = .007		
Right corticospinal tracts	*r* = 0.19	*r* = 0.49	-1.31	0.39
	*q* = 0.48	*q* = 0.03		
Left anterior cingulum	*r* = -0.14 *q* = 0.65	*r* = -0.19 *q* = 0.54	0.2	0.94
Right anterior cingulum	*r* = 0.06 *q* = 0.88	*r* = 0.11 *q* =0 .81	-0.19	0.94
Left cingulum bundle (hippocampal region)	*r* = 0.84 *q* = 1.8e-8	*r* = 0.61 *q* = 0.008	1.95	0.15
Right cingulum bundle (hippocampal region)	*r* = 0.86 *q* = 5.4e-9	*r* = 0.50 *q* = 0.07	**2.84**	**0.02**
Splenium	*r* = 0.27 *q* = 0.27	*r* = 0.02 *q* = 0.97	0.98	0.55
Genu	*r* = 0.74 *q* = 0.00001	*r* = 0.11 *q* = 0.81	**3.2**	**0.01**
Left inferior longitudinal fasciculus	*r* = 0.11 *q* = 0.81	*r* = 0.06 *q* = 0.92	0.19	0.94
Right inferior longitudinal fasciculus	*r* = -0.004 *q* = 0.99	*r* = -0.02 *q* = 0.97	-0.06	0.99
Left inferior fronto-occipital fasciculus	*r* = 0.63 *q* = 0.0009	*r* = 0.31 *q* = 0.25	1.6	0.26
Right inferior fronto-occipital fasciculus	*r* = 0.48 *q* = 0.02	*r* = 0.32 *q* = 0.44	0.73	0.74
Left superior longitudinal fasciculus	*r* = 0.37 *q* = 0.09	*r* = 0.37 *q* = 0.12	0.0	1.0
Right superior longitudinal fasciculus	*r* = 0.50 *q* = 0.02	*r* = 0.50 *q* = 0.15	0.0	1.0
Left uncinate fasciculus	*r* = 0.45 *q* = 0.03	*r* = 0.35 *q* = 0.17	0.37	0.88
Right uncinate fasciculus	*r* = 0.51 *q* = 0.01	*r* = -0.16 *q* = 0.66	**2.76**	**0.03**
Left superior longitudinal fasciculus (temporal region)	*r* = 0.28 *q* = 0.24	*r* = 0.27 *q* = 0.35	0.04	0.99
Right superior longitudinal fasciculus (temporal region)	*r* = 0.03 *q* = 0.94	*r* = -0.11 *q* = 0.81	0.54	0.81

*Values in bold show a statistically significant difference in the strength of the relationship between 22q11DS and controls.*

## DISCUSSION

This study used a whole brain and region of interest (ROI) approach to examine multiple DTI indices in 22q11DS, a neurogenetic disorder that confers significantly increased risk for the development of psychosis. Several findings emerged – some novel, while others extend upon the small body of existing 22q11DS DTI literature: (1) in comparison to controls, 22q11DS participants had reduced AD, putatively indexing axonal damage (Song et al., 2002, 2003), in multiple white matter tracts, with the strongest effect sizes observed in the SLF, a fronto-parietal tract; (2) 22q11DS participants also exhibited decreased RD, a putative marker of neuroinflammation (Colpak et al., 2014; Sasaki et al., 2014) in the splenium, SLF, and ILF; (3) there was a disrupted pattern of age-associated changes in FA and RD of the left ILF in 22q11DS, (4) increased AD in the left IFO and uncinate was associated with improved social cognition performance in both 22q11DS participants and controls, and (5) reduced AD in both the left and right IFO was associated with increased positive symptom severity in 22q11DS.

### GROUP DIFFERENCES: 22q11DS VS. TYPICALLY DEVELOPING CONTROLS

In line with prior, smaller studies we found decreased AD, a putative index of axonal disruption (Song et al., 2003), in multiple tracts in 22q11DS. Radoeva et al. (2012) also found widespread decreases in AD, including the IFO and SLF. Another group found decreased AD in the left hemisphere in 22q11DS patients relative to controls in a region that included the intersection of IFO, ILF, SLF, cingulum, and anterior thalamic radiations (ATR; Kikinis et al., 2012). Using DTI tractography, this group also identified reduced AD in the IFO and ILF in 22q11DS participants (Kikinis et al., 2013). Murine models resulting in axonal degeneration or axonal damage and/or loss have observed decreased AD (Song et al., 2003; Budde et al., 2008). Other studies in animal models have found that reduced diameter of axonal bundles is also associated with reduced AD (Schwartz et al., 2005; Harsan et al., 2006). Thus, the reduced AD observed in 22q11DS may reflect reduced diameter of axonal bundles, axonal degeneration, damage, and/or loss. To our knowledge, however, white matter microstructure has not been directly investigated in mouse models of 22q11DS.

It is important to note that axonal damage does not occur in isolation. Song et al. (2003) found that degradation to the myelin sheath occurred *after* axonal degeneration, and Harsan et al. (2006) used a transgenic mouse that expressed a virus in oligodendrocytes, also resulting in oligodendrocyte apoptosis and demyelination (in addition to decreased axonal diameter). Supporting these findings, we found that measures of AD and RD were significantly related to each other in multiple regions, in both 22q11DS patients and controls.

Surprisingly, in both our ROI and whole-brain analyses, we also found decreased RD in 22q11DS, which was not observed in two prior 22q11DS studies that examined specific sub-components of FA (Kikinis et al., 2012; Radoeva et al., 2012). However, more recently, Perlstein et al. (2014) used white matter tractography to examine three tracts of interest (i.e., ALIC, fornix, and uncinate) and found that decreased RD drove the observed increases in FA in bilateral regions of the ALIC in 22q11DS. Recent studies in human populations have found decreased RD to be associated with neuroinflammation, as decreased RD in multiple brain regions has been found in individuals with autoimmune disease (Colpak et al., 2014) and history of concussion (Sasaki et al., 2014). Decreased RD has also been observed in a rodent model of thrombotic stroke (induced by hypoxia-ischemia); in this study, decreased RD correlated with swelling of myelin sheaths (Shereen et al., 2011). Upon further investigation, the authors found that cerebral hypoxia-ischemia rapidly induced oxidative stress in oligodendrocytes, resulting in swelling of myelin and compression of axoplasma (Shereen et al., 2011). Others have found that smaller axonal diameter was associated with decreased RD in the corpus callosum of the rat brain (Barazany et al., 2009). On the other hand, increased RD has been observed in shiverer mice, who have thin, loosely packed, or absent myelin sheaths, but intact axonal integrity (Song et al., 2002). Similarly, a cuprizone mouse model, which results in oligodendrocyte loss followed by demyelination, also showed increased RD (Song et al., 2005). Thus, the significant decreases in RD that we observed in human subjects with 22q11DS could potentially reflect swelling of myelin sheaths and/or compression of axoplasma, reduced axonal diameter, and/or increased myelination.

In our whole-brain analyses, those with 22q11DS had higher FA in several regions, including the posterior limb of the internal capsule, the corona radiata, the body of the corpus callosum, and a small region of the left SLF. These findings are unexpected, given that the majority of DTI studies to date have found decreased FA in 22q11DS patients (e.g., Barnea-Goraly et al., 2003; Sundram et al., 2010; Radoeva et al., 2012; Villalon-Reina et al., 2013). However, our findings do replicate increases in FA previously observed in 22q11DS in the corona radiata (Sundram et al., 2010) and SLF (Simon et al., 2005). Additionally, somewhat consistent with our findings of increased FA in the body of the corpus callosum, Barnea-Goraly et al. (2003) found increased FA in the genu and the splenium of the corpus callosum in 22q11DS. Furthermore, the findings of increased FA in 22q11DS are consistent with evidence from other neurogenetic syndromes involving anomalous neurodevelopment, i.e., William’s syndrome, (Hoeft et al., 2007; Arlinghaus et al., 2011; Haas et al., 2013).

Although the cellular mechanisms underlying increased FA in 22q11DS in these regions are unknown, our findings were likely driven by a combination of decreased AD and RD. Regional increases in FA in children with other neurodevelopmental disorders has been observed, and the authors of these studies have offered a number of speculations as to causal mechanisms at the cellular level, such as: decreases in axonal branching (Hoeft et al., 2007), fewer obliquely oriented fibers (Cheng et al., 2011), flattened fibers, enabling the increased density of white matter (Bode et al., 2011), or decreases in fiber crossing (Arlinghaus et al., 2011). Genes located within the 22q11 locus, such as the Reticulon 4 receptor gene (RTN4R), which is associated with axonal growth inhibition (Fournier et al., 2001), may contribute to white matter microstructural abnormalities in 22q11DS. However, given that, to our knowledge, no mouse models of 22q11DS have examined white matter pathology, the precise contributing cellular mechanisms remain unknown.

In contrast, our ROI analyses did not indicate higher FA in 22q11DS participants relative to controls within the tracts investigated. The ROI approach, which averages all the voxels within a specific region, may have masked the increased FA identified in specific tracts (i.e., posterior limb of the internal capsule, superior and posterior corona radiate, body of the corpus callosum, small portion of the left SLF) in the whole-brain approach. For example, the SLF is a large ROI and the small portion of increased FA that we identified in the whole-brain analysis could have been obscured when the average FA was calculated for that ROI. Additionally, the ROIs used in this study did not cover the entire mean skeleton obtained (e.g., the body of the corpus callosum was not an identified ROI), and thus such regions were not included in the ROI analyses. However, despite the lack of significantly increased FA in the ROI analyses, we did find decreased AD and RD in regions that produced comparable results to the whole brain findings. These findings also highlight the importance of using separate measures of diffusivity to examine white matter abnormalities in patient groups, as in other brain disorders (Nir et al., 2013).

Although the number of published DWI studies in 22q11DS is small (11 publications as of June 2014), the findings have varied, due in part to the methodological differences and sample characteristics. For example, many of the studies did not align the *b*0 image to a T1-weighted structural scan to correct for EPI induced artifacts, which result in spatial distortions that could substantially change the results. Another reason for these discrepant findings may be due to difficulties encountered when registering DWI data (Smith et al., 2006), particularly when the shape of tracts differs between groups. Though the optimal type of registration is not agreed upon (Smith et al., 2006; Schwarz et al., 2014), differences in registration methods used can also result in substantially different results. Furthermore, customization of ROIs may help in obtaining accurate results in clinical populations, particularly those with neurogenetic conditions. For example, editing the ROIs obtained from the Johns Hopkins University atlas to fit our mean skeleton was necessary for proper placement of the ROIs entirely within white matter. Thus, we suggest that future studies investigate how the selected ROIs map onto one’s data prior to running analyses, and adjust the ROIs if necessary. To our knowledge, this is not standard practice in DTI studies, but has been performed in published manuscripts by our collaborators (Karlsgodt et al., 2009).

### AGE ASSOCIATED DISRUPTIONS IN WHITE MATTER MICROSTRUCTURE IN 22q11DS

We also found age-related disruptions in white matter microstructure of the left ILF in those with 22q11DS, as 22q11DS participants failed to show the age-associated increases in FA observed in typically developing controls. Notably, a similar finding has been observed in youth at CHR for psychosis, who failed to show typical age-related FA increases in the medial temporal lobe (Karlsgodt et al., 2009). Our finding appears to be driven by an age-associated disruption of RD in the ILF of 22q11DS participants. Typically developing adolescents show decreases in RD as they age, which is believed to reflect increased myelination (Asato et al., 2010; Simmonds et al., 2014); however, our results suggest that 22q11DS participants do not show this pattern of decreasing RD with age. Although the age^∗^group interaction only reached significance within this tract, several other regions, including the SLF, appeared to follow a similar pattern, and these results approached significance. There may be an atypical neurodevelopmental trajectory of white matter microstructure in 22q11DS; one hypothesis is that those with 22q11DS undergo “precocious maturation,” with myelination occurring before the period of adolescence, closing the window for heightened brain plasticity during adolescence and young adulthood. This pattern of increased early myelination has been observed in mice who underwent early life stress (i.e., being weaned from their mothers at an early age (Ono et al., 2008; Kikusui and Mori, 2009). Notably, like patients with 2q211DS, these mice displayed increased anxious behaviors (Ono et al., 2008). Thus, 22q11DS participants may have “early” myelination, resulting in increased FA at an earlier age and failure to show the typical increase in adolescence and young adulthood. Equally plausible is the possibility of delayed myelination; for example, structural neuroanatomic studies of 22q11DS have found a pattern of delayed cortical maturation has been observed in children with attention deficit hyperactivity disorder (Shaw et al., 2007). This possibility is consistent with a previous structural neuroimaging (Schaer et al., 2009) study and a proton spectroscopy study (Shashi et al., 2012b) of 22q11DS participants. For example, FA may increase at a later age in those with 22q11DS relative to typically developing youth. However, these hypotheses need to be tested with prospective longitudinal investigations, which are currently in progress.

Additionally, the wide variability of findings in the 22q11DS literature may be at least partly due to developmental changes taking place on white matter microstructure. Development of white matter, particularly fronto-temporal and limbic connections, continues to take place during adolescence and young adulthood (Bava et al., 2010; Giorgio et al., 2010; Lebel et al., 2012; Simmonds et al., 2014). Notably, white matter changes in adolescence parallel the development of cognitive and social-affective processes during this sensitive period (Blakemore and Choudhury, 2006; Choudhury et al., 2006), which may be relevant to the development of psychosis (Paus et al., 2008). The majority of previous DTI studies of 22q11DS did not covary for age when examining group differences (Barnea-Goraly et al., 2003; Simon et al., 2005; Radoeva et al., 2012; Kikinis et al., 2013, 2012) and none of these studies examined how age-associated white matter changes may be disrupted in 22q11DS.

### ASSOCIATION OF WHITE MATTER MICROSTRUCTURE WITH SOCIAL COGNITION PERFORMANCE IN 22q11DS AND CONTROLS

Greater axonal coherence (i.e., higher AD) in the IFO and uncinate was associated with improved social cognition in both 22q11DS patients and healthy controls. Similar results have been reported in a combined analysis of 22q11DS participants and controls: increased AD in the right hemisphere of the SLF, the posterior corona radiata, and IFO was related to better social skills (as measured by the socialization subdomain of the Vineland Adaptive Behavior Scales, Radoeva et al., 2012). The IFO is the longest association tract in the brain and has multiple connections between the occipital, temporal, and frontal lobes (Martino et al., 2010; Sarubbo et al., 2013). It has been hypothesized that the IFO is crucial for integrating information between physically distant brain regions (Sarubbo et al., 2013), which is essential for integrating social information from one’s environment and responding appropriately. Furthermore, damage to the IFO results in impairment in emotion recognition (Philippi et al., 2009), highlighting the important role that this tract plays in facilitating connections between visual processing and emotion-related cortical regions (i.e., visual cortex to orbitofrontal cortex).

To our knowledge, this study is the first to relate higher AD in fronto-limbic tracts (i.e., uncinate fasciculus) to better social cognition in both controls and 22q11DS participants. The uncinate connects the amygdala to the anterior temporal lobe and orbitofrontal cortex (Ghashghaei and Barbas, 2002; Ghashghaei et al., 2007) and is believed to play a role in the interaction between emotion and cognition (Barbas, 2000). This white matter tract has been associated with the following socially related functions: evaluation of stimuli, social reward processing, and higher-level emotional meaning of concepts (for a review, Von Der Heide et al., 2013). Thus, it is not surprising that we see that greater axonal coherence in this tract is associated with improved social cognition performance in both 22q11DS participants and controls.

### ASSOCIATION OF WHITE MATTER MICROSTRUCTURE WITH POSITIVE SYMPTOMS IN 22q11DS

To our knowledge, this is the first study to relate positive symptoms and lower AD in 22q11DS. Importantly, decreased AD in bilateral IFO was related to increased positive symptom severity in 22q11DS, is consistent with the previously noted association between AD in the left IFO with improved social cognition performance in 22q11DS participants. In a prior behavioral study, the same social cognition task (i.e., TASIT) was the most significantly related to psychotic symptomatology in 22q11DS, when compared to other measures of social and non-social cognition (Jalbrzikowski et al., 2012). Disrupted axonal coherence in the IFO may underlie the social cognition impairment and psychotic symptoms in 22q11DS, suggesting a common mechanism of brain disturbance.

### RELATIONSHIP OF FINDINGS TO IDIOPATHIC SCHIZOPHRENIA

Multiple studies in patients with idiopathic schizophrenia have consistently demonstrated that disruption in white matter microstructure in multiple regions is driven by increased radial, not axial, diffusivity, which authors interpret as indicating that white matter dysfunction in idiopathic schizophrenia is primarily driven by demyelination, rather than axonal damage (Seal et al., 2008; Levitt et al., 2012; Lee et al., 2013). This hypothesis is supported by the post-mortem histopathology literature, which shows disturbances in the function and structure of oligodendrocytes, brain cells responsible for the myelination of axons (Davis et al., 2003; Walterfang et al., 2006). Thus, as postulated by Kikinis et al. (2012), it is possible that white matter pathology associated with psychosis in 22qDS is driven by different neuropathological mechanisms relative to idiopathic psychosis. Nevertheless, such perturbations of structural connectivity between brain regions critical for social processing may lead to downstream commonalities in their phenotypic effects. Finally, these findings are not consistent with the majority of the idiopathic schizophrenia literature, other studies have found increased FA in white matter microstructure in other neurogenetic disorders, such as Williams’ syndrome (Hoeft et al., 2007; Arlinghaus et al., 2011; Haas et al., 2013), which may be attributable to a decrease in normal amount of branching in these cortical tracts, leading to less fiber crossing and thus resulting in increased FA (Hoeft et al., 2007).

### RELATIONSHIP OF FINDINGS TO EXISTING 22q11DS NEUROIMAGING LITERATURE

This work complements the existing structural and functional neuroimaging work in 22q11DS. For example, structural abnormalities of the corpus callosum in 22q11DS have been detected in multiple studies(Shashi et al., 2004, 2012a; Baker et al., 2011), and we see white matter abnormalities in the corpus callosum (increased FA in the body of the corpus callosum, decreased RD and AD in the splenium, and decreased AD in the genu). However, to our knowledge, the relationship between white matter volumetric and DTI measures in 22q11DS has not yet been examined. Additionally, a combined structural and diffusion MRI study of 22q11DS found an overall global loss of connectivity (6%) in 22q11DS participants compared to controls (Ottet et al., 2013b), and another study of resting state functional connectivity showed deficits in long range connectivity in 22q11DS youth (Schreiner et al., 2014); these findings are consistent with the AD deficits seen in long range association tracts (i.e., ILF, IFO, SLF) that we observed in 22q11DS. Reduced frontal–temporal functional connectivity has also been observed in 22q11DS (Ottet et al., 2013a) and we observed reduced AD in the uncinate fasciculus, a frontal–medial temporal tract. In the future, it will be important to conduct studies that incorporate multiple neuroimaging modalities (e.g., DTI, fMRI) in 22q11DS to better understand how these findings inform each other.

The observed relationship between reduced AD and increased psychotic symptom severity in 22q11DS also extends upon the existing literature on relationships between neuroimaging measures and the psychosis phenotype in 22q11DS. Two recent studies have found white matter microstructure abnormalities in the cingulum bundle (increased FA and decreased RD), anterior limb of the internal capsule (increased right FA, decreased left RD), and uncinate fasciculus (decreased bilateral RD) have been associated with increased psychotic symptoms in 22q11DS (Kates et al., 2014; Perlstein et al., 2014). To our knowledge, ours is the first study to identify a significant relationship between decreased AD in bilateral regions of the IFO and increased psychotic symptom severity in 22q11DS. Taken together, these findings provide support for the notion that structural dysconnectivity is particularly relevant to the psychosis phenotype in 22q11DS. Furthermore, the relationships previously observed between decreased RD and psychotic symptoms in 22q11DS (Kates et al., 2014; Perlstein et al., 2014), paired with our findings of decreased RD in 22q11DS overall, suggest that decreased RD may also be pathological.

### LIMITATIONS

Several limitations of this study should be noted. First, given our cross-sectional design we were unable to investigate changes in DTI measures over time as predictors of subsequent development of psychotic symptoms in 22q11DS. In the future, it will be critical to incorporate a longitudinal approach, as studies of structural neuroanatomic predictors of psychosis have found change over time to be a strong predictor of symptom development, in both 22q11DS (Kates et al., 2011) and idiopathic psychosis (Sun et al., 2009; Takahashi et al., 2009). Secondly, established DTI measures may not be equipped to measure the complexity of increased fiber crossing that is hypothesized to occur as one ages (Riffert et al., 2014). One specific drawback to this methodology is that, within one voxel, only one primary direction of diffusion can be calculated, despite the fact that there are many axons within a voxel (Basser et al., 2000; Pierpaoli et al., 2001). For example, in a region that has many different fibers crossing in different directions, the mean of the primary direction is calculated, which may result in a pattern of globally reduced FA in this region, even if FA is high in these different crossing fibers. Thus, it is possible that with increasing age controls are developing a more complex pattern of fiber crossing than those with 22q11DS. However, the limitations in scan resolution make it difficult to test this hypothesis. Other types of methodologies, such as q-ball imaging, measure diffusion without making assumptions about the underlying white matter microstructure (Tuch, 2004). However, this imaging technique prolongs scan time, which is not always feasible for clinical populations. Therefore, advanced DTI techniques to quantify the complexity of fiber crossing are currently in development (Riffert et al., 2014). Finally, scans were conducted on two different scanners; although these scanners were identical (Siemens 3 Tesla Tim Trio) and the proportion of patients and controls was similar across scanners, we nevertheless observed significant differences as a function of scanner location (Supplementary Table S2). Nevertheless, these differences were systematic and consistent across regions, as FA was consistently higher on one scanner, whereas AD and RD were consistently lower on this scanner. Thus, we covaried for scanner site in all analyses.

### FUTURE DIRECTIONS

The current study sets a foundation to develop future multi-modal neuroimaging biomarker studies in 22q11DS. The white matter pathways that we found to be associated with social cognition and psychosis (i.e., IFO) connect to a gray matter region, the medial orbitofrontal cortex, variation in which we have also found to be associated with psychosis in 22q11DS (Jalbrzikowski et al., 2013). Thus, to better understand how neuropathophysiological mechanisms are related to both social impairment and psychotic symptoms in 22q11DS, it will be important to examine relationships between measures of structural white matter connectivity and gray matter thickness, and in turn, how these measures relate to behavior, in both healthy individuals and those with 22q11DS. Furthermore, future investigations in larger samples of white matter microstructure in 22q11DS in relation to genetic pathways will be particularly informative given that multiple genes within the deleted region are associated with neuronal development (Maynard et al., 2003). Specifically, well-validated bioinformatics approaches (Zhang and Horvath, 2005) have been developed, allowing us to identify pathways or modules of gene expression related to psychosis in 22q11DS patients, and relate these molecular features to neuroimaging and clinical data, thus connecting genes to brain to behavior, and setting up future studies to more thoroughly assess causality and mechanism both in humans and in animal models.

## Conflict of Interest Statement

The authors declare that the research was conducted in the absence of any commercial or financial relationships that could be construed as a potential conflict of interest.

## References

[B1] Alexander A. L. Lee J. E. Lazar M. Field A. S. (2007). Diffusion tensor imaging of the brain. *Neurotherapeutics* 4 316–329 10.1016/j.nurt.2007.05.011 17599699 PMC2041910

[B2] Arlinghaus L. R. Thornton-Wells T. A. Dykens E. M. Anderson A. W. (2011). Alterations in diffusion properties of white matter in Williams syndrome. *Magn. Reson. Imaging* 29 1165–1174 10.1016/j.mri.2011.07.012 21907520 PMC3199335

[B3] Asato M. R. Terwilliger R. Woo J. Luna B. (2010). White matter development in adolescence: a DTI Study. *Cereb. Cortex* 20 2122–2131 10.1093/cercor/bhp282 20051363 PMC2923214

[B4] Baker K. Chaddock C. A. Baldeweg T. Skuse D. (2011). Neuroanatomy in adolescents and young adults with 22q11 deletion syndrome: comparison to an IQ-matched group. *Neuroimage* 55 491–499 10.1016/j.neuroimage.2010.12.041 21184831

[B5] Barazany D. Basser P. J. Assaf Y. (2009). *In vivo* measurement of axon diameter distribution in the corpus callosum of rat brain. *Brain* 132 1210–1220 10.1093/brain/awp042 19403788 PMC2677796

[B6] Barbas H. (2000). Connections underlying the synthesis of cognition, memory, and emotion in primate prefrontal cortices. *Brain Res. Bull.* 52 319–330 10.1016/S0361-9230(99)00245-2 10922509

[B7] Barnea-Goraly N. Menon V. Krasnow B. Ko A. Reiss A. Eliez S. (2003). Investigation of white matter structure in velocardiofacial syndrome: a diffusion tensor imaging study. *Am. J. Psychiatry* 160 1863–1869 10.1176/appi.ajp.160.10.1863 14514502

[B8] Basser P. J. Pajevic S. Pierpaoli C. Duda J. Aldroubi A. (2000). In vivo fiber tractography using DT-MRI data. *Magn. Reson. Med.* 44 625–632 10.1002/1522-2594(200010)44:4<625::AID-MRM17>3.0.CO;2-O 11025519

[B9] Bava S. Thayer R. Jacobus J. Ward M. Jernigan T. L. Tapert S. F. (2010). Longitudinal characterization of white matter maturation during adolescence. *Brain Res.* 1327 38–46 10.1016/j.brainres.2010.02.066 20206151 PMC2854176

[B10] Blakemore S.-J. Choudhury S. (2006). Development of the adolescent brain: implications for executive function and social cognition. *J. Child Psychol. Psychiatry* 47 296–312 10.1111/j.1469-7610.2006.01611.x 16492261

[B11] Bloemen O. J. N. de Koning M. B. Schmitz N. Nieman D. H. Becker H. E. de Haan L. (2010). White-matter markers for psychosis in a prospective ultra-high-risk cohort. *Psychol. Med.* 40 1297–1304 10.1017/S0033291709991711 19895720

[B12] Bode M. K. Mattila M. L. Kiviniemi V. Rahko J. Moilanen I. Ebeling H. (2011). White matter in autism spectrum disorders – evidence of impaired fiber formation. *Acta Radiol.* 52 1169–1174 10.1258/ar.2011.110197 22101385

[B13] Budde M. D. Kim J. H. Liang H.-F. Russell J. H. Cross A. H. Song S.-K. (2008). Axonal injury detected by in vivo diffusion tensor imaging correlates with neurological disability in a mouse model of multiple sclerosis. *NMR Biomed.* 21 589–597 10.1002/nbm.1229 18041806 PMC2602834

[B14] Budde M. D. Xie M. Cross A. H. Song S.-K. (2009). Axial diffusivity is the primary correlate of axonal injury in the experimental autoimmune encephalomyelitis spinal cord: a quantitative pixelwise analysis. *J. Neurosci.* 29 2805–2813 10.1523/JNEUROSCI.4605-08.2009 19261876 PMC2673458

[B15] Burns J. Job D. Bastin M. E. Whalley H. Macgillivray T. Johnstone E. C. (2003). Structural disconnectivity in schizophrenia: a diffusion tensor magnetic resonance imaging study. *Br. J. Psychiatry* 182 439–443 10.1192/bjp.182.5.439 12724248

[B16] Carletti F. Woolley J. B. Bhattacharyya S. Perez-Iglesias R. Fusar-Poli P. Valmaggia L. (2012). Alterations in white matter evident before the onset of psychosis. *Schizophr. Bull.* 38 1170–1179 10.1093/schbul/sbs053 22472474 PMC3494044

[B17] Carter C. S. Barch D. M. Gur R. Gur R. Pinkham A. Ochsner K. (2009). CNTRICS final task selection: social cognitive and affective neuroscience-based measures. *Schizophr. Bull.* 35 153–162 10.1093/schbul/sbn157 19011231 PMC2643972

[B18] Cheng Y. Chou K.-H. Fan Y.-T. Lin C.-P. (2011). ANS: aberrant neurodevelopment of the social cognition network in adolescents with autism spectrum disorders. *PLoS ONE* 6:e18905 10.1371/journal.pone.0018905 PMC3082537 21541322

[B19] Choudhury S. Blakemore S.-J. Charman T. (2006). Social cognitive development during adolescence. *Soc. Cogn. Affect. Neurosci.* 1 165–174 10.1093/scan/nsl024 18985103 PMC2555426

[B20] Clemm von Hohenberg C. Pasternak O. Kubicki M. Ballinger T. Vu M.-A. Swisher T. (2014). White matter microstructure in individuals at clinical high risk of psychosis: a whole-brain diffusion tensor imaging study. *Schizophr. Bull.* 40 895–903 10.1093/schbul/sbt079 23737549 PMC4059424

[B21] Colpak A. I. Kurne A. T. Oguz K. K. Has A. C. Dolgun A. Kansu T. (2014). White matter involvement beyond the optic nerves in CRION as assessed by diffusion tensor imaging. *Int. J. Neurosci.* 10.3109/00207454.2014.896912 [Epub ahead of print]. 24588222

[B22] da Silva Alves F. Schmitz N. Bloemen O. van der Meer J. Meijer J. Boot E. (2011). Schizophrenia Research. *Schizophr. Res.* 132 75–83 10.1016/j.schres.2011.07.017 21831603

[B23] Davis K. L. Stewart D. G. Friedman J. I. Buchsbaum M. Harvey P. D. Hof P. R. (2003). White matter changes in schizophrenia: evidence for myelin-related dysfunction. *Arch. Gen. Psychiatry* 60 443–456 10.1001/archpsyc.60.5.443 12742865

[B24] Ellison-Wright I. Bullmore E. (2009). Meta-analysis of diffusion tensor imaging studies in schizophrenia. *Schizophr. Res.* 108 3–10 10.1016/j.schres.2008.11.021 19128945

[B25] Federspiel A. Begre S. Kiefer C. Schroth G. Strik W. K. Dierks T. (2006). Alterations of white matter connectivity in first episode schizophrenia. *Neurobiol. Dis.* 22 702–709 10.1016/j.nbd.2006.01.015 16624566

[B26] First M. B. Spitzer R. L. Gibbon M. Williams J. (2002). *Structured Clinical Interview for DSM-IV-TR Axis I Disorders, Research Version, Patient Edition (SCID-I/P)*. New York, NY: Biometrics Research, New York State Psychiatric Institute.

[B27] Fitzsimmons J. Hamoda H. M. Swisher T. Terry D. Rosenberger G. Seidman L. J. (2014). Diffusion tensor imaging study of the fornix in first episode schizophrenia and in healthy controls. *Schizophr. Res.* 156 157–160 10.1016/j.schres.2014.04.022 24837684 PMC4080801

[B28] Fournier A. E. GrandPre T. Strittmatter S. M. (2001). Identification of a receptor mediating Nogo-66 inhibition of axonal regeneration. *Nature* 409 341–346 10.1038/35053072 11201742

[B29] Friedman J. I. Tang C. Carpenter D. Buchsbaum M. Schmeidler J. Flanagan L. (2008). Diffusion tensor imaging findings in first-episode and chronic schizophrenia patients. *Am. J. Psychiatry* 165 1024–1032 10.1176/appi.ajp.2008.07101640 18558643

[B30] Gasparotti R. Valsecchi P. Carletti F. Galluzzo A. Liserre R. Cesana B. (2009). Reduced fractional anisotropy of corpus callosum in first-contact, antipsychotic drug-naive patients with schizophrenia. *Schizophr. Res.* 108 41–48 10.1016/j.schres.2008.11.015 19103476

[B31] Ghashghaei H. T. Barbas H. (2002). Pathways for emotion: interactions of prefrontal and anterior temporal pathways in the amygdala of the rhesus monkey. *Neuroscience* 115 1261–1279 10.1016/S0306-4522(02)00446-3 12453496

[B32] Ghashghaei H. T. Hilgetag C. C. Barbas H. (2007). Sequence of information processing for emotions based on the anatomic dialogue between prefrontal cortex and amygdala. *Neuroimage* 34 905–923 10.1016/j.neuroimage.2006.09.046 17126037 PMC2045074

[B33] Giorgio A. Watkins K. E. Chadwick M. James S. Winmill L. Douaud G. (2010). Longitudinal changes in grey and white matter during adolescence. *Neuroimage* 49 94–103 10.1016/j.neuroimage.2009.08.003 19679191

[B34] Goldenberg P. C. Calkins M. E. Richard J. McDonald-McGinn D. Zackai E. Mitra N. (2012). Computerized neurocognitive profile in young people with 22q11.2 deletion syndrome compared to youths with schizophrenia and at-risk for psychosis. *Am. J. Med. Genet. B. Neuropsychiatr. Genet.* 159B, 87–93 10.1002/ajmg.b.32005 22170773 PMC3272485

[B35] Gothelf D. Feinstein C. Thompson T. Gu E. Penniman L. Van Stone E. (2007). Risk factors for the emergence of psychotic disorders in adolescents with 22q11.2 deletion syndrome. *Am. J. Psychiatry* 164 663–669 10.1176/appi.ajp.164.4.663 17403981

[B36] Green M. F. Bearden C. E. Cannon T. D. Fiske A. P. Hellemann G. S. Horan W. P. (2012). Social cognition in schizophrenia, part 1: performance across phase of Illness. *Schizophr. Bull.* 38 854–864 10.1093/schbul/sbq171 21345917 PMC3406534

[B37] Green T. Gothelf D. Glaser B. Debbané M. Frisch A. Kotler M. (2009). Psychiatric disorders and intellectual functioning throughout development in velocardiofacial (22q11.2 deletion) syndrome. *J. Am. Acad. Child Adolesc. Psychiatry* 48 1060–1068 10.1097/CHI.0b013e3181b76683 19797984

[B38] Guo W. Liu F. Liu Z. Gao K. Xiao C. Chen H. (2012). Right lateralized white matter abnormalities in first-episode, drug-naive paranoid schizophrenia. *Neurosci. Lett.* 531 5–9 10.1016/j.neulet.2012.09.033 23022507

[B39] Gur R. E. Yi J. J. McDonald-McGinn D. M. Tang S. X. Calkins M. E. Whinna D. (2014). Neurocognitive development in 22q11.2 deletion syndrome: comparison with youth having developmental delay and medical comorbidities. *Mol. Psychiatry* 19 1205–1211 10.1038/mp.2013.189 PMC4450860 24445907

[B40] Haas B. W. Barnea-Goraly N. Sheau K. E. Yamagata B. Ullas S. Reiss A. L. (2013). Altered microstructure within social-cognitive brain networks during childhood in Williams syndrome. *Cereb. Cortex* 2796–2806 10.1093/cercor/bht135 23709644 PMC4207879

[B41] Harsan L. A. Poulet P. Guignard B. Steibel J. Parizel N. de Sousa P. L. (2006). Brain dysmyelination and recovery assessment by noninvasive in vivo diffusion tensor magnetic resonance imaging. *J. Neurosci. Res.* 83 392–402 10.1002/jnr.20742 16397901

[B42] Henze R. Brunner R. Thiemann U. Parzer P. Klein J. Resch F. (2012). White matter alterations in the corpus callosum of adolescents with first-admission schizophrenia. *Neurosci. Lett.* 513 178–182 10.1016/j.neulet.2012.02.032 22373786

[B43] Hoeft F. Barnea-Goraly N. Haas B. W. Golarai G. Ng D. Mills D. (2007). More is not always better: increased fractional anisotropy of superior longitudinal fasciculus associated with poor visuospatial abilities in Williams syndrome. *J. Neurosci.* 27 11960–11965 10.1523/JNEUROSCI.3591-07.2007 17978036 PMC6673356

[B44] Jalbrzikowski M. Carter C. Senturk D. Chow C. Hopkins J. M. Green M. F. (2012). Schizophrenia research. *Schizophr. Res.* 142 99–107 10.1016/j.schres.2012.10.007 23122739 PMC3714207

[B45] Jalbrzikowski M. Jonas R. Senturk D. Patel A. Chow C. Green M. F. (2013). Structural abnormalities in cortical volume, thickness, and surface area in 22q11.2 microdeletion syndrome: relationship with psychotic symptoms. *Neuroimage Clin.* 3 405–415 10.1016/j.nicl.2013.09.013 PMC3814944 24273724

[B46] Jones D. K. Knosche T. R. Turner R. (2013). White matter integrity, fiber count, and other fallacies: the do“s and don”ts of diffusion MRI. *Neuroimage* 73 239–254 10.1016/j.neuroimage.2012.06.081 22846632

[B47] Karlsgodt K. H. Niendam T. A. Bearden C. E. Cannon T. D. (2009). White matter integrity and prediction of social and role functioning in subjects at ultra-high risk for psychosis. *Biol. Psychiatry* 66 562–569 10.1016/j.biopsych.2009.03.013 19423081 PMC2805703

[B48] Karlsgodt K. H. Sun D. Jimenez A. M. Lutkenhoff E. S. Willhite R. van Erp T. G. M. (2008). Developmental disruptions in neural connectivity in the pathophysiology of schizophrenia. *Dev. Psychopathol.* 20 1297–1327 10.1017/S095457940800062X 18838043

[B49] Kates W. R. Antshel K. M. Faraone S. V. Fremont W. P. Higgins A. M. Shprintzen R. J. (2011). Neuroanatomic predictors to prodromal psychosis in velocardiofacial syndrome (22q11.2 Deletion Syndrome): a longitudinal study. *Biol. Psychiatry* 69 945–952 10.1016/j.biopsych.2010.10.027 PMC3081962 21195387

[B50] Kates W. R. Olszewski A. K. Gnirke M. H. Kikinis Z. Nelson J. Antshel K. (2014). White matter microstructural abnormalities of the cingulum bundle in youths with 22q11.2 deletion syndrome: associations with medication, neuropsychological function, and prodromal symptoms of psychosis. *Schizophr. Res.* 10.1016/j.schres.2014.07.010 [Epub ahead of print]. PMC4277733 25066496

[B51] Kawashima T. Nakamura M. Bouix S. Kubicki M. Salisbury D. F. Westin C.-F. (2009). Uncinate fasciculus abnormalities in recent onset schizophrenia and affective psychosis: a diffusion tensor imaging study. *Schizophr. Res.* 110 119–126 10.1016/j.schres.2009.01.014 19328656 PMC2749228

[B52] Kikinis Z. Asami T. Bouix S. Finn C. T. Ballinger T. Tworog-Dube E. (2012). Reduced fractional anisotropy and axial diffusivity in white matter in 22q11.2 deletion syndrome: a pilot study. *Schizophr. Res.* 141 35–39 10.1016/j.schres.2012.06.032 22863550 PMC3462006

[B53] Kikinis Z. Makris N. Finn C. T. Bouix S. Lucia D. Coleman M. J. (2013). Genetic contributions to changes of fiber tracts of ventral visual stream in 22q11.2 deletion syndrome. *Brain Imaging Behav.* 7 316–325 10.1007/s11682-013-9232-5 PMC3796180 23612843

[B54] Kikusui T. Mori Y. (2009). Behavioural and neurochemical consequences of early weaning in rodents. *J. Neuroendocrinol.* 21 427–431 10.1111/j.1365-2826.2009.01837.x 19207810

[B55] Kitis O. Ozalay O. Zengin E. B. Haznedaroglu D. Eker M. C. Yalvac D. (2012). Reduced left uncinate fasciculus fractional anisotropy in deficit schizophrenia but not in non-deficit schizophrenia. *Psychiatry Clin. Neurosci.* 66 34–43 10.1111/j.1440-1819.2011.02293.x 22250608

[B56] Kohler C. G. Bilker W. Hagendoorn M. Gur R. E. Gur R. C. (2000). Emotion recognition deficit in schizophrenia: association with symptomatology and cognition. *Biol. Psychiatry* 48 127–136 10.1016/S0006-3223(00)00847-7 10903409

[B57] Kong X. Ouyang X. Tao H. Liu H. Li L. Zhao J. (2011). Complementary diffusion tensor imaging study of the corpus callosum in patients with first-episode and chronic schizophrenia. *J. Psychiatry Neurosci.* 36 120–125 10.1503/jpn.100041 21138657 PMC3044195

[B58] Kuswanto C. N. Teh I. Lee T.-S. Sim K. (2012). Diffusion tensor imaging findings of white matter changes in first episode schizophrenia: a systematic review. *Clin. Psychopharmacol. Neurosci.* 10 13–24 10.9758/cpn.2012.10.1.13 23429992 PMC3569158

[B59] Lebel C. Gee M. Camicioli R. Wieler M. Martin W. Beaulieu C. (2012). Diffusion tensor imaging of white matter tract evolution over the lifespan. *Neuroimage* 60 340–352 10.1016/j.neuroimage.2011.11.094 22178809

[B60] Lee S.-H. Kubicki M. Asami T. Seidman L. J. Goldstein J. M. Mesholam-Gately R. I. (2013). Extensive white matter abnormalities in patients with first-episode schizophrenia: a diffusion tensor imaging (DTI) study. *Schizophr. Res.* 143 231–238 10.1016/j.schres.2012.11.029 23290268 PMC4354799

[B61] Leow A. Huang S.-C. Geng A. Becker J. Davis S. Toga A. (2005). Inverse consistent mapping in 3D deformable image registration: its construction and statistical properties. *Inf. Process. Med. Imaging* 19 493–503. 17354720 10.1007/11505730_41

[B62] Leow A. D. Zhu S. Zhan L. McMahon K. de Zubicaray G. I. Meredith M. (2009). The tensor distribution function. *Magn. Reson. Med.* 61 205–214 10.1002/mrm.21852 19097208 PMC2770429

[B63] Levitt J. J. Alvarado J. L. Nestor P. G. Rosow L. Pelavin P. E. McCarley R. (2012). Fractional anisotropy and radial diffusivity: diffusion measures of white matter abnormalities in the anterior limb of the internal capsule in schizophrenia. *Schizophr. Res.* 136 55–62 10.1016/j.schres.2011.09.009 22019073

[B64] Liu X. Lai Y. Wang X. Hao C. Chen L. Zhou Z. (2013). Reduced white matter integrity and cognitive deficit in never-medicated chronic schizophrenia: a diffusion tensor study using TBSS. *Behav. Brain Res.* 252 157–163 10.1016/j.bbr.2013.05.061 23747517

[B65] Luck D. Malla A. K. Joober R. Lepage M. (2010). Disrupted integrity of the fornix in first-episode schizophrenia. *Schizophr. Res.* 119 61–64 10.1016/j.schres.2010.03.027 20409692

[B66] Martino J. Brogna C. Robles S. G. Vergani F. Duffau H. (2010). Anatomic dissection of the inferior fronto-occipital fasciculus revisited in the lights of brain stimulation data. *Cortex* 46 691–699 10.1016/j.cortex.2009.07.015 19775684

[B67] Maynard T. M. Haskell G. T. Peters A. Z. Sikich L. Lieberman J. A. LaMantia A. S. (2003). A comprehensive analysis of 22q11 gene expression in the developing and adult brain. *Proc. Natl. Acad. Sci. U.S.A.* 100 14433–14438 10.1073/pnas.2235651100 14614146 PMC283609

[B68] McDonald S. Bornhofen C. Shum D. Long E. Saunders C. Neulinger K. (2006). Reliability and validity of the awareness of social inference test (TASIT): a clinical test of social perception. *Disabil. Rehabil.* 28 1529–1542 10.1080/09638280600646185 17178616

[B69] McDonald S. Flanagan S. Rollins J. Kinch J. (2003). TASIT: a new clinical tool for assessing social perception after traumatic brain injury. *J. Head Trauma Rehabil.* 18 219–238 10.1097/00001199-200305000-00001 12802165

[B70] McGlashan T. Miller T. J. Woods S. W. Hoffman R. E. Davidson L. (2001). “A scale for the McGlashan assessment of prodromal symptoms and states,” in *Early Intervention in Psychotic Disorders* eds Miller T. J. Mednick S. A. McGlashan T. Liberger J. Johannessen J. O. (Norwell, MA: Kluwer Academic Publishers), 135–149.

[B71] Meyer S. E. Bearden C. E. Lux S. R. Gordon J. L. Johnson J. K. O’Brien M. (2005). The psychosis prodrome in adolescent patients viewed through the lens of DSM-IV. *J. Child Adolesc. Psychopharmacol.* 15 434–451 10.1089/cap.2005.15.434 16092909

[B72] Miller T. J. McGlashan T. H. Rosen J. L. Cadenhead K. Cannon T. Ventura J. (2003). Prodromal assessment with the structured interview for prodromal syndromes and the scale of prodromal symptoms: predictive validity, interrater reliability, and training to reliability. *Schizophr. Bull.* 29 703–715 10.1093/oxfordjournals.schbul.a007040 14989408

[B73] Mori S. Wakana S. Nagae-Poetscher L. M. van Zijl P. (2005). *MRI Atlas of Human White Matter.* Amsterdam: Elsevier. 10.1148/radiol.2301021640 14645885

[B74] Mori S. Zhang J. (2006). Principles of diffusion tensor imaging and its applications to basic neuroscience research. *Neuron* 51 527–539 10.1016/j.neuron.2006.08.012 16950152

[B75] Murphy K. C. Jones L. A. Owen M. J. (1999). High rates of schizophrenia in adults with velo-cardio-facial syndrome. *Arch. Gen. Psychiatry* 56 940–945 10.1001/archpsyc.56.10.940 10530637

[B76] Nestor P. G. Kubicki M. Nakamura M. Niznikiewicz M. Levitt J. J. Shenton M. E. (2013). Neuropsychological variability, symptoms, and brain imaging in chronic schizophrenia. *Brain Imaging Behav.* 7 68–76 10.1007/s11682-012-9193-0 23011383

[B77] Nir T. M. Jahanshad N. Villalon-Reina J. E. Toga A. W. Jack C. R. Weiner M. W. (2013). Effectiveness of regional DTI measures in distinguishing Alzheimer’s disease, MCI, and normal aging. *Neuroimage Clin.* 3 180–195 10.1016/j.nicl.2013.07.006 24179862 PMC3792746

[B78] Ono M. Kikusui T. Sasaki N. Ichikawa M. Mori Y. Murakami-Murofushi K. (2008). Early weaning induces anxiety and precocious myelination in the anterior part of the basolateral amygdala of male Balb/c mice. *Neuroscience* 156 1103–1110 10.1016/j.neuroscience.2008.07.078 18790016

[B79] Ottet M.-C. Schaer M. Cammoun L. Schneider M. Debbané M. Thiran J.-P. (2013a). Reduced fronto-temporal and limbic connectivity in the 22q11.2 deletion syndrome: vulnerability markers for developing schizophrenia? *PLoS ONE* 8:e58429 10.1371/journal.pone.0058429 PMC3606218 23533586

[B80] Ottet M.-C. Schaer M. Debbané M. Cammoun L. Thiran J.-P. Eliez S. (2013b). Graph theory reveals dysconnected hubs in 22q11DS and altered nodal efficiency in patients with hallucinations. *Front. Hum. Neurosci.* 7:402 10.3389/fnhum.2013.00402 PMC3763187 24046733

[B81] Paus T. Keshavan M. Giedd J. N. (2008). Why do many psychiatric disorders emerge during adolescence? *Nat. Rev. Neurosci.* 9 947–957 10.1038/nrn2513 19002191 PMC2762785

[B82] Perlstein M. D. Chohan M. R. Coman I. L. Antshel K. M. Fremont W. P. Gnirke M. H. (2014). White matter abnormalities in 22q11.2 deletion syndrome: preliminary associations with the Nogo-66 receptor gene and symptoms of psychosis. *Schizophr. Res.* 152 117–123 10.1016/j.schres.2013.11.015 24321711 PMC3909835

[B83] Peters B. D. de Haan L. Dekker N. Blaas J. Becker H. E. Dingemans P. M. (2008). White matter fibertracking in first-episode schizophrenia, schizoaffective patients and subjects at ultra-high risk of psychosis. *Neuropsychobiology* 58 19–28 10.1159/000154476 18781087

[B84] Peters B. D. Karlsgodt K. H. (2014). White matter development in the early stages of psychosis. *Schizophr. Res.* 10.1016/j.schres.2014.05.021 [Epub ahead of print]. PMC4250450 24893908

[B85] Philippi C. L. Mehta S. Grabowski T. Adolphs R. Rudrauf D. (2009). Damage to association fiber tracts impairs recognition of the facial expression of emotion. *J. Neurosci.* 29 15089–15099 10.1523/JNEUROSCI.0796-09.2009 19955360 PMC2819193

[B86] Pierpaoli C. Barnett A. Pajevic S. Chen R. Penix L. R. Virta A. (2001). Water diffusion changes in Wallerian degeneration and their dependence on white matter architecture. *Neuroimage* 13 1174–1185 10.1006/nimg.2001.0765 11352623

[B87] Pinkham A. E. Penn D. L. Perkins D. O. Lieberman J. (2003). Implications for the neural basis of social cognition for the study of schizophrenia. *Am. J. Psychiatry* 160 815–824 10.1176/appi.ajp.160.5.815 12727681

[B88] Price G. Cercignani M. Parker G. J. M. Altmann D. R. Barnes T. R. E. Barker G. J. (2008). White matter tracts in first-episode psychosis: a DTI tractography study of the uncinate fasciculus. *Neuroimage* 39 949–955 10.1016/j.neuroimage.2007.09.012 17988894 PMC2387199

[B89] Pulver A. E. Nestadt G. Goldberg R. Shprintzen R. J. Lamacz M. Wolyniec P. S. (1994). Psychotic illness in patients diagnosed with velo-cardio-facial syndrome and their relatives. *J. Nerv. Ment. Dis.* 182 476–478 10.1097/00005053-199408000-00010 8040660

[B90] R Core Team (2014). *R: A Language and Environment for Statistical Computing*. Vienna: R Foundation for Statistical Computing Available at: http://www.R-project.org/

[B91] Radoeva P. D. Coman I. L. Antshel K. M. Fremont W. McCarthy C. S. Kotkar A. (2012). Atlas-based white matter analysis in individuals with velo-cardio-facial syndrome (22q11.2 deletion syndrome) and unaffected siblings. *Behav. Brain Funct.* 8 38 10.1186/1744-9081-8-38 PMC3533822 22853778

[B92] Riffert T. W. Schreiber J. Anwander A. Knosche T. R. (2014). Beyond fractional anisotropy: extraction of bundle-specific structural metrics from crossing fiber models. *Neuroimage* 100C, 176–191 10.1016/j.neuroimage.2014.06.015 24936681

[B93] Roalf D. R. Ruparel K. Verma R. Elliott M. A. Gur R. E. Gur R. C. (2013). White matter organization and neurocognitive performance variability in schizophrenia. *Schizophr. Res.* 143 172–178 10.1016/j.schres.2012.10.014 23148898 PMC3540127

[B94] Roddy S. Tiedt L. Kelleher I. Clarke M. C. Murphy J. Rawdon C. (2012). Facial emotion recognition in adolescents with psychotic-like experiences: a school-based sample from the general population. *Psychol. Med.* 42 2157–2166 10.1017/S0033291712000311 22370095

[B95] Samartzis L. Dima D. Fusar-Poli P. Kyriakopoulos M. (2014). White matter alterations in early stages of schizophrenia: a systematic review of diffusion tensor imaging studies. *J. Neuroimaging* 24 101–110 10.1111/j.1552-6569.2012.00779.x 23317110

[B96] Sarubbo S. De Benedictis A. Maldonado I. L. Basso G. Duffau H. (2013). Frontal terminations for the inferior fronto-occipital fascicle: anatomical dissection, DTI study and functional considerations on a multi-component bundle. *Brain Struct. Funct.* 218 21–37 10.1007/s00429-011-0372-3 22200882

[B97] Sasaki T. Pasternak O. Mayinger M. Muehlmann M. Savadjiev P. Bouix S. (2014). Hockey concussion education project, part 3. White matter microstructure in ice hockey players with a history of concussion: a diffusion tensor imaging study. *J. Neurosurg.* 120 882–890 10.3171/2013.12.JNS132092 PMC4863636 24471841

[B98] Schaer M. Debbané M. Bach Cuadra M. Ottet M.-C. Glaser B. Thiran J.-P. (2009). Deviant trajectories of cortical maturation in 22q11.2 deletion syndrome (22q11DS): a cross-sectional and longitudinal study. *Schizophr. Res.* 115 182–190 10.1016/j.schres.2009.09.016 19836927

[B99] Scheel M. Prokscha T. Bayerl M. Gallinat J. Montag C. (2013). Myelination deficits in schizophrenia: evidence from diffusion tensor imaging. *Brain Struct. Funct.* 218 151–156 10.1007/s00429-012-0389-2 22327232

[B100] Schenkel L. S. Pavuluri M. N. Herbener E. S. Harral E. M. Sweeney J. A. (2007). Facial emotion processing in acutely ill and euthymic patients with pediatric bipolar disorder. *J. Am. Acad. Child Adolesc. Psychiatry* 46 1070–1079 10.1097/chi.0b013e3180600fd6 17667485

[B101] Schneider M. Debbané M. Bassett A. S. Chow E. W. C. Fung W. L. A. van den Bree M. B. M. (2014). Psychiatric disorders from childhood to adulthood in 22q11.2 deletion syndrome: results from the international consortium on brain and behavior in 22q11.2 deletion syndrome. *Am. J. Psychiatry* 171 627–639 10.1176/appi.ajp.2013.13070864 24577245 PMC4285461

[B102] Schreiner M. J. Karlsgodt K. H. Uddin L. Q. Chow C. Congdon E. Jalbrzikowski M. (2014). Default mode network connectivity and reciprocal social behavior in 22q11.2 deletion syndrome. *Soc. Cogn. Affect. Neurosci.* 9 1261–1267 10.1093/scan/nst114 PMC4158365 23912681

[B103] Schwarz C. G. Reid R. I. Gunter J. L. Senjem M. L. Przybelski S. A. Zuk S. M. (2014). Improved DTI registration allows voxel-based analysis that outperforms tract-based spatial statistics. *Neuroimage* 94 65–78 10.1016/j.neuroimage.2014.03.026 24650605 PMC4137565

[B104] Schwartz E. D. Cooper E. T. Fan Y. Jawad A. F. Chin C.-L. Nissanov J. (2005). MRI diffusion coefficients in spinal cord correlate with axon morphometry. *Neuroreport* 16 73–76 10.1097/00001756-200501190-00017 15618894

[B105] Seal M. L. Yucel M. Fornito A. Wood S. J. Harrison B. J. Walterfang M. (2008). Abnormal white matter microstructure in schizophrenia: a voxelwise analysis of axial and radial diffusivity. *Schizophr. Res.* 101 106–110 10.1016/j.schres.2007.12.489 18262770

[B106] Shashi V. Francis A. Hooper S. R. Kranz P. G. Zapadka M. Schoch K. (2012a). Increased corpus callosum volume in children with chromosome 22q11.2 deletion syndrome is associated with neurocognitive deficits and genetic polymorphisms. *Eur. J. Hum. Genet.* 20 1051–1057 10.1038/ejhg.2012.138 PMC3449066 22763378

[B107] Shashi V. Veerapandiyan A. Keshavan M. S. Zapadka M. Schoch K. Kwapil T. R. (2012b). Altered development of the dorsolateral prefrontal cortex in chromosome 22q11.2 deletion syndrome: an in vivo proton spectroscopy study. *Biol. Psychiatry* 72 684–691 10.1016/j.biopsych.2012.04.023 PMC3440535 22633947

[B108] Shashi V. Muddasani S. Santos C. C. Berry M. N. Kwapil T. R. Lewandowski E. (2004). Abnormalities of the corpus callosum in nonpsychotic children with chromosome 22q11 deletion syndrome. *Neuroimage* 21 1399–1406 10.1016/j.neuroimage.2003.12.004 15050565

[B109] Shaw P. Eckstrand K. Sharp W. Blumenthal J. Lerch J. P. Greenstein D. (2007). Attention-deficit/hyperactivity disorder is characterized by a delay in cortical maturation. *Proc. Natl. Acad. Sci. U.S.A.* 104 19649–19654 10.1073/pnas.0707741104 18024590 PMC2148343

[B110] Shereen A. Nemkul N. Yang D. Adhami F. Dunn R. S. Hazen M. L. (2011). Ex vivo diffusion tensor imaging and neuropathological correlation in a murine model of hypoxia-ischemia-induced thrombotic stroke. *J. Cereb. Blood Flow Metab.* 31 1155–1169 10.1038/jcbfm.2010.212 21139628 PMC3070976

[B111] Simmonds D. J. Hallquist M. N. Asato M. Luna B. (2014). Developmental stages and sex differences of white matter and behavioral development through adolescence: a longitudinal diffusion tensor imaging (DTI) study. *Neuroimage* 92 356–368 10.1016/j.neuroimage.2013.12.044 24384150 PMC4301413

[B112] Simon T. J. Ding L. Bish J. P. McDonald-McGinn D. M. Zackai E. H. Gee J. (2005). Volumetric, connective, and morphologic changes in the brains of children with chromosome 22q11.2 deletion syndrome: an integrative study. *Neuroimage* 25 169–180 10.1016/j.neuroimage.2004.11.018 15734353

[B113] Smith S. M. Nichols T. E. (2009). Threshold-free cluster enhancement: addressing problems of smoothing, threshold dependence and localisation in cluster inference. *Neuroimage* 44 83–98 10.1016/j.neuroimage.2008.03.061 18501637

[B114] Smith S. M. Jenkinson M. Johansen-Berg H. Rueckert D. Nichols T. E. Mackay C. E. (2006). Tract-based spatial statistics: voxelwise analysis of multi-subject diffusion data. *Neuroimage* 31 1487–1505 10.1016/j.neuroimage.2006.02.024 16624579

[B115] Song S.-K. Sun S.-W. Ju W.-K. Lin S.-J. Cross A. H. Neufeld A. H. (2003). Diffusion tensor imaging detects and differentiates axon and myelin degeneration in mouse optic nerve after retinal ischemia. *Neuroimage* 20 1714–1722 10.1016/j.neuroimage.2003.07.005 14642481

[B116] Song S.-K. Sun S.-W. Ramsbottom M. J. Chang C. Russell J. Cross A. H. (2002). Dysmyelination revealed through MRI as increased radial (but unchanged axial) diffusion of water. *Neuroimage* 17 1429–1436 10.1006/nimg.2002.1267 12414282

[B117] Song S.-K. Yoshino J. Le T. Q. Lin S.-J. Sun S.-W. Cross A. H. (2005). Demyelination increases radial diffusivity in corpus callosum of mouse brain. *Neuroimage* 26 132–140 10.1016/j.neuroimage.2005.01.028 15862213

[B118] Sun D. Phillips L. Velakoulis D. Yung A. Mcgorry P. D. Wood S. J. (2009). Progressive brain structural changes mapped as psychosis develops in “at risk” individuals. *Schizophr. Res.* 108 85–92 10.1016/j.schres.2008.11.026 19138834 PMC2670732

[B119] Sundram F. Campbell L. E. Azuma R. Daly E. Bloemen O. J. N. Barker G. J. (2010). White matter microstructure in 22q11 deletion syndrome: a pilot diffusion tensor imaging and voxel-based morphometry study of children and adolescents. *J. Neurodevelop. Disord.* 2 77–92 10.1007/s11689-010-9043-6 PMC3164036 22127856

[B120] Szeszko P. R. Ardekani B. A. Ashtari M. Kumra S. Robinson D. G. Sevy S. (2005). White matter abnormalities in first-episode schizophrenia or schizoaffective disorder: a diffusion tensor imaging study. *Am. J. Psychiatry* 162 602–605 10.1176/appi.ajp.162.3.602 15741480

[B121] Szeszko P. R. Robinson D. G. Ashtari M. Vogel J. Betensky J. Sevy S. (2008). Clinical and neuropsychological correlates of white matter abnormalities in recent onset schizophrenia. *Biol. Psychiatry* 33 976–984 10.1038/sj.npp.1301480 17581532

[B122] Takahashi T. Wood S. J. Yung A. R. Soulsby B. Mcgorry P. D. Suzuki M. (2009). Progressive gray matter reduction of the superior temporal gyrus during transition to psychosis. *Arch. Gen. Psychiatry* 66 366–376 10.1001/archgenpsychiatry.2009.12 19349306

[B123] Thomason M. E. Thompson P. M. (2011). Diffusion imaging, white matter, and psychopathology. *Annu. Rev. Clin. Psychol.* 7 63–85 10.1146/annurev-clinpsy-032210-104507 21219189

[B124] Tuch D. S. (2004). Q-ball imaging. *Magn. Reson. Med.* 52 1358–1372 10.1002/mrm.20279 15562495

[B125] Villalon-Reina J. Jahanshad N. Beaton E. Toga A. W. Thompson P. M. Simon T. J. (2013). White matter microstructural abnormalities in girls with chromosome 22q11.2 deletion syndrome, Fragile X or Turner syndrome as evidenced by diffusion tensor imaging. *Neuroimage* 81 441–454 10.1016/j.neuroimage.2013.04.028 PMC3947617 23602925

[B126] Von Der Heide R. J. Skipper L. M. Klobusicky E. Olson I. R. (2013). Dissecting the uncinate fasciculus: disorders, controversies and a hypothesis. *Brain* 136 1692–1707 10.1093/brain/awt094 23649697 PMC3673595

[B127] Wakana S. Jiang H. Nagae-Poetscher L. M. van Zijl P. C. M. Mori S. (2004). Fiber tract-based atlas of human white matter anatomy. *Radiology* 230 77–87 10.1148/radiol.2301021640 14645885

[B128] Walterfang M. Wood S. J. Velakoulis D. Pantelis C. (2006). Neuropathological, neurogenetic and neuroimaging evidence for white matter pathology in schizophrenia. *Neurosci. Biobehav. Rev.* 30 918–948 10.1016/j.neubiorev.2006.02.001 16580728

[B129] Yan H. Tian L. Yan J. Sun W. Liu Q. Zhang Y.-B. (2012). Functional and anatomical connectivity abnormalities in cognitive division of anterior cingulate cortex in schizophrenia. *PLoS ONE* 7:e45659 10.1371/journal.pone.0045659 PMC3458074 23049832

[B130] Zhan L. Leow A. D. Zhu S. Baryshev M. Toga A. W. McMahon K. L. (2009). A novel measure of fractional anisotropy based on the tensor distribution function. *Med. Image Comput. Comput. Assist. Interv.* 12 845–852. 20426067 10.1007/978-3-642-04268-3_104

[B131] Zhang B. Horvath S. (2005). A general framework for weighted gene co-expression network analysis. *Stat. Appl. Genet. Mol. Biol.* 4 17 10.2202/1544-6115.1128 16646834

