# Supplementary material for: Altered white matter microstructure is associated with social cognition and psychotic symptoms in 22q11.2 microdeletion syndrome
Source: Front Behav Neurosci. 2014 Nov 11;8:393. doi: 10.3389/fnbeh.2014.00393 (PMC4227518; doi:10.3389/fnbeh.2014.00393)

Supplementary Figure 1. Plotted below are the overall raw mean FA values for regions that showed significant differences between 22q11DS and controls in the whole-brain analysis (i.e., right posterior limb of the internal capsule, right superior and posterior corona radiata, body of corpus callosum, and a small portion of the left SLF).

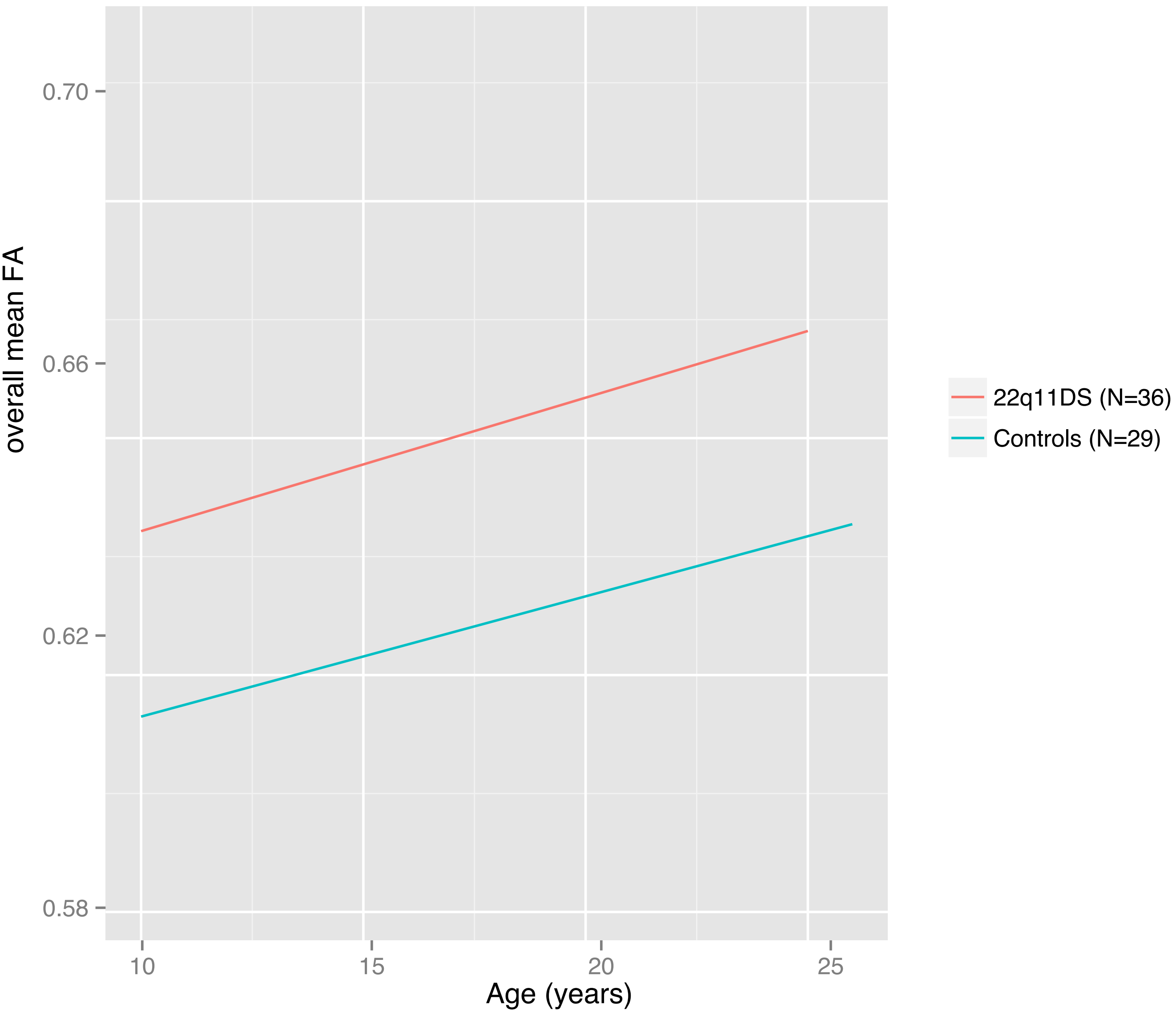

Supplement: Supplementary file 1 [file Image_1.PDF]
