# Supplementary material for: Altered white matter microstructure is associated with social cognition and psychotic symptoms in 22q11.2 microdeletion syndrome
Source: Front Behav Neurosci. 2014 Nov 11;8:393. doi: 10.3389/fnbeh.2014.00393 (PMC4227518; doi:10.3389/fnbeh.2014.00393)
Supplement: Supplementary file 2 [file Table_1.DOC]

Supplementary Table 1. Comparison of age (A) and gender (B) of 22q11DS participants and controls between and within scanner location (Brain Mapping Center=BMC, Center for Cognitive Neuroscience= CCN).

A.

|  | 22q11DS (N=36) | Controls (N=29) |  |
| --- | --- | --- | --- |
| BMC (years, +/- SD) | 15.9 (4.1) | 15.4 (4.5) | *p*=.78 |
| CCN (years, +/- SD) | 16.5 (4.4) | 15.6 (2.7) | *p*=.91 |
|  | *p*=.65 | *p*=.90 |  |

B.

|  | 22q11DS (N=36) | Controls (N=29) |  |
| --- | --- | --- | --- |
| BMC (N, % female) | 9 (60%) | 10 (63%) | *p*=.89 |
| CCN (N, % female) | 16 (76%) | 4 (31%) | *p*=.009 |
|  | *p*=.30 | *p*=.09 |  |
