# Supplementary material for: Altered white matter microstructure is associated with social cognition and psychotic symptoms in 22q11.2 microdeletion syndrome
Source: Front Behav Neurosci. 2014 Nov 11;8:393. doi: 10.3389/fnbeh.2014.00393 (PMC4227518; doi:10.3389/fnbeh.2014.00393)
Supplement: Supplementary file 3 [file Table_2.DOC]

Supplementary Table 2: Differences in regions of interest within white matter tracts as a function of scanner location.

|  |  | Fractional Anisotropy | | | Axial Diffusivity | | | Radial Diffusivity | | |
| --- | --- | --- | --- | --- | --- | --- | --- | --- | --- | --- |
|  |  | F-statistic | *q*-value | 🡩 or 🡫 in BMC scanner | F-statistic | q-value | 🡩 or 🡫  in BMC scanner | F-statistic | *q*-value | 🡩 or 🡫 in BMC scanner |
| Anterior Thalamic Radiations | LH | 11.7 | .003 | **🡩** | 14.8 | .001 | **🡫** | 17.9 | .0005 | **🡫** |
|  | RH | 42.5 | .000001 | **🡩** | 21.2 | .0002 | **🡫** | 49.9 | .000001 | **🡫** |
| Corticospinal Tracts | LH | 13.5 | .003 | **🡩** | 7.4 | .02 | **🡫** | 26.7 | .00004 | **🡫** |
|  | RH | 21.2 | .0002 | **🡩** | 5.8 | .04 | **🡫** | 29.3 | .00002 | **🡫** |
| Anterior Cingulum | LH | 3.7 | .11 |  | 1.4 | .33 |  | 9.5 | .009 | **🡫** |
|  | RH | 2.8 | .16 |  | 7.9 | .02 | **🡫** | 11.2 | .003 | **🡫** |
| Cingulum Bundle (hippocampal region) | LH | .04 | .90 |  | .6 | .52 |  | .42 | .61 |  |
|  | RH | 3.1 | .14 |  | 7.5 | .02 | **🡫** | 7.3 | .02 | **🡫** |
| Corpus  Callosum | Splenium | .02 | .91 |  | 11.9 | .003 | **🡫** | .63 | .52 |  |
|  | Genu | 2.8 | .16 |  | 3.9 | .09 |  | 4.9 | .06 |  |
| Inferior Frontal-  occipital Fasciculus | LH | 2.7 | .17 |  | 1.0 | .41 |  | 4.2 | .09 |  |
|  | RH | 15.8 | .0009 | **🡩** | 7.4 | .02 | **🡫** | 23.9 | .00008 | **🡫** |
| Inferior Longitudinal  Fasciculus | LH | 1.3 | .34 |  | .15 | .79 |  | 1.7 | .27 |  |
|  | RH | 5.9 | .04 | **🡩** | .11 | .82 |  | 4.6 | .09 |  |
| Superior Longitudinal  Fasciculus | LH | .87 | .45 |  | 3.4 | .12 |  | .03 | .9 |  |
|  | RH | 5.4 | .04 | **🡩** | 13.9 | .0009 | **🡫** | 16.5 | .0006 | **🡫** |
| Uncinate  Fasciculus | LH | .03 | .9 |  | 2.7 | .17 |  | 2.4 | .19 |  |
|  | RH | 7.4 | .02 | **🡩** | 2.2 | .2 |  | 15.3 | .0009 | **🡫** |
| Superior Longitudinal | LH | .3 | .67 |  | .04 | .9 |  | .007 | .93 |  |
| Fasciculus (temporal region) | RH | 14.4 | .001 | **🡩** | 1.7 | .2727273 |  | 29.2 | .00001 | **🡫** |
