# Supplementary material for: Altered white matter microstructure is associated with social cognition and psychotic symptoms in 22q11.2 microdeletion syndrome
Source: Front Behav Neurosci. 2014 Nov 11;8:393. doi: 10.3389/fnbeh.2014.00393 (PMC4227518; doi:10.3389/fnbeh.2014.00393)
Supplement: Supplementary file 4 [file Table_3.DOC]

Supplementary Table 3. Difffusion tensor imaging results for ROIs within white matter tracts in participants with 22q11DS versus typically developing controls.

|  |  | Fractional Anisotropy | | | | Axial Diffusivity | | | | Radial Diffusivity | | | |
| --- | --- | --- | --- | --- | --- | --- | --- | --- | --- | --- | --- | --- | --- |
| Region | Hemi-sphere | F-statistic | *q*-value | Effect size ( partial *η^2^*) | 🡩 or 🡫 in 22q11DS | F-statistic | *q*-value | Effect size ( partial *η^2)^* | 🡩 or 🡫 in 22q11DS | F-statistic | *q*-value | Effect size ( partial *η^2^*) | 🡩 or 🡫 in 22q11DS |
| Anterior Thalamic Radiations | LH | 0.1 | 0.84 | .002 |  | 3.8 | 0.12 | .06 |  | 0.0 | 0.95 | .000 |  |
|  | RH | 1.9 | 0.28 | .03 |  | 2.5 | 0.21 | .04 |  | 1.9 | 0.28 | .03 |  |
| Corticospinal | LH | 0.1 | 0.84 | .002 |  | 5.2 | 0.06 | .08 |  | 2.5 | 0.22 | .04 |  |
| Tracts | RH | 0.0 | 0.98 | .000 |  | 5.7 | 0.05 | .09 |  | 2.5 | 0.22 | .04 |  |
| Anterior Cingulum | LH | 0.3 | 0.75 | .004 |  | 10.0 | 0.01 | .14 | 🡫 | 4.0 | 0.11 | .06 |  |
|  | RH | 0.3 | 0.75 | .005 |  | 20.6 | 0.0003 | .26 | 🡫 | 2.1 | 0.26 | .03 |  |
| Cingulum Bundle | LH | 9.8 | 0.01 | .14 | 🡫 | 0.1 | 0.91 | .001 |  | 4.9 | 0.07 | .08 |  |
| (hippocampal region) | RH | 1.8 | 0.29 | .03 |  | 0.8 | 0.57 | .01 |  | 0.4 | 0.71 | .007 |  |
| Corpus  Callosum | Splenium | 0.4 | 0.71 | .006 |  | 26.4 | 0.00005 | .31 | 🡫 | 8.7 | 0.02 | .13 | 🡫 |
|  | Genu | 0.1 | 0.84 | .002 |  | 11.3 | 0.007 | .16 | 🡫 | 3.1 | 0.17 | .05 |  |
| Inferior Frontal-  occipital Fasciculus | LH | 0.1 | 0.84 | .002 |  | 25.7 | 0.00005 | .30 | 🡫 | 5.5 | 0.06 | .08 |  |
|  | RH | 0.4 | 0.71 | .007 |  | 19.1 | 0.0004 | .24 | 🡫 | 2.6 | 0.22 | .04 |  |
| Inferior Longitudinal  Fasciculus | LH | 0.3 | 0.73 | .006 |  | 19.2 | 0.0004 | .24 | 🡫 | 8.1 | 0.02 | .12 | 🡫 |
|  | RH | 0.0 | 0.98 | .000 |  | 30.2 | 0.00002 | .34 | 🡫 | 6.5 | 0.04 | .09 | 🡫 |
| Superior Longitudinal  Fasciculus | LH | 0.4 | 0.71 | .007 |  | 40.6 | 0.000001 | .40 | 🡫 | 9.6 | 0.01 | .14 | 🡫 |
|  | RH | 0.3 | 0.75 | .004 |  | 39.9 | 0.000001 | .40 | 🡫 | 12.1 | 0.005 | .17 | 🡫 |
| Uncinate  Fasciculus | LH | 0.0 | 0.95 | .000 |  | 12.1 | 0.005 | .17 | 🡫 | 5.4 | 0.06 | .08 |  |
|  | RH | 3.2 | 0.16 | .051 |  | 0.0 | 0.95 | .00 |  | 5.8 | 0.06 | .09 |  |
| Superior Longitudinal | LH | 0.0 | 0.99 | .000 |  | 5.3 | 0.06 | .08 |  | 1.3 | 0.40 | .02 |  |
| Fasiculus (temporal region) | RH | 0.6 | 0.67 | .009 |  | 13.9 | 0.003 | .19 | 🡫 | 10.6 | 0.009 | .15 | 🡫 |
