# Supplementary material for: Altered white matter microstructure is associated with social cognition and psychotic symptoms in 22q11.2 microdeletion syndrome
Source: Front Behav Neurosci. 2014 Nov 11;8:393. doi: 10.3389/fnbeh.2014.00393 (PMC4227518; doi:10.3389/fnbeh.2014.00393)
Supplement: Supplementary file 5 [file Table_4.DOC]

Supplementary Table 4. Difffusion tensor imaging results for ROIs within white matter tracts in participants with 22q11.2 deletion syndrome versus typically developing controls at each scanner location. Regions highlighted in green were statistically significant in the overall group analysis and continue to show significance (p<.05) or a trend towards significance (p<.10) at both scanner locations.

|  |  | Fractional Anisotropy | | | | Axial Diffusivity | | | | Radial Diffusivity | | | |
| --- | --- | --- | --- | --- | --- | --- | --- | --- | --- | --- | --- | --- | --- |
|  |  | BMC | | CCN | | BMC | | CCN | | BMC | | CCN | |
| Region | Hemi-sphere | F-statistic | *p*-value | F-statistic | *p*-value | F-statistic | *p*-value | F-statistic | *p*-value | F-statistic | *p*-value | F-statistic | *p*-value |
| Anterior Thalamic Radiations | LH | 0.1 | .7 | 0.04 | .84 | 1.6 | 0.22 | 3.1 | 0.089 | 0.02 | .9 | 0.2 | 0.7 |
|  | RH | 0.5 | 0.5 | 1.3 | 0.3 | 2.4 | 0.13 | 1.1 | 0.3 | 0.9 | .3 | 1.1 | 0.3 |
| Corticospinal | LH | 0.02 | 0.9 | 0.02 | 0.9 | 2.4 | 0.13 | 2.2 | 0.2 | 1.3 | 0.3 | 1.6 | 0.2 |
| Tracts | RH | 0.5 | 0.5 | 0.5 | 0.5 | 4.2 | 0.05 | 1.2 | 0.3 | 0.4 | 0.5 | 3.4 | 0.08 |
| Anterior Cingulum | LH | 0.7 | 0.4 | 0.2 | 0.7 | 3.8 | 0.08 | 10.8 | .003 | 3.7 | 0.07 | 0.8 | 0.4 |
|  | RH | 0.1 | .7 | 0.2 | 0.7 | 7.1 | 0.01 | 12.3 | 0.001 | 0.5 | 0.5 | 1.6 | 0.2 |
| Cingulum Bundle | LH | 6.1 | 0.02 | 3.1 | 0.08 | 0.05 | 0.8 | .04 | 0.9 | 2.9 | 0.09 | 1.9 | 0.2 |
| (hippocampal region) | RH | 0.04 | 0.8 | 3.0 | 0.09 | 0.08 | 0.8 | 0.2 | 0.7 | 0.05 | 0.8 | 0.8 | 0.4 |
| Corpus  Callosum | Splenium | 3.8 | 0.06 | 1.0 | 0.32 | 10.9 | 0.003 | 15.4 | 0.0005 | 11.4 | 0.002 | 0.6 | 0.4 |
|  | Genu | 1.6 | 0.2 | 0.3 | 0.6 | 4.8 | 0.036 | 9.9 | 0.004 | 5.1 | 0.03 | 0.5 | 0.5 |
| Inferior Frontal-  occipital Fasciculus | LH | 0.2 | 0.6 | 0.9 | 0.4 | 15.0 | 0.001 | 12.3 | 0.001 | 4.9 | 0.03 | 1.6 | 0.2 |
|  | RH | 0.3 | 0.6 | 3.6 | 0.07 | 7.5 | 0.01 | 12.2 | 0.001 | 2.4 | 0.1 | 0.2 | 0.6 |
| Inferior Longitudinal  Fasciculus | LH | 3.7 | 0.07 | 1.6 | 0.2 | 6.9 | 0.014 | 12.1 | 0.002 | 8.0 | 0.009 | 1.2 | 0.3 |
|  | RH | 0.4 | 0.5 | 0.9 | 0.3 | 16.7 | 0.0003 | 14.2 | 0.001 | 4.8 | 0.04 | 1.6 | 0.2 |
| Superior Longitudinal  Fasciculus | LH | 3.2 | 0.09 | 0.5 | 0.5 | 16.1 | 0.0004 | 22.5 | 0.00005 | 10.2 | 0.004 | 1.6 | 0.2 |
|  | RH | 2.3 | 0.14 | 1.1 | 0.3 | 11.9 | 0.002 | 31.39 | 0.000004 | 8.7 | 0.007 | 3.5 | 0.07 |
| Uncinate  Fasciculus | LH | 0.03 | 0.87 | 0.4 | 0.5 | 7.8 | 0.010 | 3.8 | 0.06 | 2.6 | 0.11 | 5.2 | 0.03 |
|  | RH | 3.4 | 0.08 | 0.6 | 0.4 | 0.15 | 0.7 | 0.11 | 0.7 | 1.9 | 0.18 | 4.7 | 0.04 |
| Superior Longitudinal | LH | 1.0 | 0.33 | 0.4 | 0.5 | 1.9 | 0.2 | 3.0 | 0.09 | 2.4 | 0.13 | 0.08 | 0.8 |
| Fasiculus (temporal region) | RH | 0.07 | .8 | 1.1 | 0.3 | 8.5 | 0.007 | 2.97 | 0.10 | 4.1 | 0.05 | 7.8 | 0.009 |
