# Supplementary material for: Altered white matter microstructure is associated with social cognition and psychotic symptoms in 22q11.2 microdeletion syndrome
Source: Front Behav Neurosci. 2014 Nov 11;8:393. doi: 10.3389/fnbeh.2014.00393 (PMC4227518; doi:10.3389/fnbeh.2014.00393)
Supplement: Supplementary file 6 [file Table_5.DOC]

Supplementary Table 5*.

A) Correlations between measures of AD in white matter regions of interest (ROIs) and positive symptoms in individuals with 22q11DS, after regressing out the effects of age, sex, scanner location, and Full Scale IQ score.

B) Correlations between measures of AD in white matter ROIs and social cognition measures in individuals with 22q11DS, after regressing out the effects of age, sex, scanner location, and Full Scale IQ score.

C) Correlations between measures of AD in white matter ROIs and social cognition variables in controls, after regressing out the effects of age, sex, scanner location, and Full Scale IQ score.

A.

| Region | Total Positive Symptoms (SIPS) |
| --- | --- |
| LH IFO AD | *r*= -.38 *p*=.02 |
| RH IFO AD | *r*= -.41 *p*=.02 |

B.

| Region | TASIT | ER40 |
| --- | --- | --- |
| LH IFO AD | *r*=.39 *p*=.02 | *r*=.37 *p*=.03 |
| LH uncinate AD | *r*=.78 *p*=2E-8 | *r*=.32 *p*=.05 |

C.

| Region | TASIT |
| --- | --- |
| LH IFO AD | *r*=.40 *p*=.05 |
| LH uncinate AD | *r*=.41 *p*=.03 |
| LH ILF AD | *r*=.21  *p*=.29 |
| RH ILF AD | *r*=.13  *p*=.52 |
| LH SLF AD | *r*=.50 *p*=.008 |
| RH SLF AD | *r*=.43 *p*=.03 |

*All IQ-adjusted analyses were conducted only for regions that showed significant relationships between DTI indices and social cognition variables
